# Sociodemographic Differences in COVID-19 Pandemic Experiences Among Families in the United States

**DOI:** 10.1001/jamanetworkopen.2023.30495

**Published:** 2023-08-23

**Authors:** Kaja Z. LeWinn, Leonardo Trasande, Andrew Law, Courtney K. Blackwell, Traci A. Bekelman, Jessica A. Arizaga, Alexis A. Sullivan, Theresa M. Bastain, Carrie V. Breton, Margaret R. Karagas, Amy J. Elliott, Catherine J. Karr, Kecia N. Carroll, Anne L. Dunlop, Lisa A. Croen, Amy E. Margolis, Akram N. Alshawabkeh, Jose F. Cordero, Anne Marie Singh, Christine M. Seroogy, Daniel J. Jackson, Robert A. Wood, Tina V. Hartert, Young Shin Kim, Cristiane S. Duarte, Julie B. Schweitzer, Barry M. Lester, Cynthia T. McEvoy, Thomas G. O’Connor, Emily Oken, Nicole Bornkamp, Eric D. Brown, Christina A. Porucznik, Assiamira Ferrara, Carlos A. Camargo, Qi Zhao, Jody M. Ganiban, Lisa P. Jacobson

**Affiliations:** 1 Department of Psychiatry and Behavioral Sciences, University of California San Francisco; 2 Department of Pediatrics, New York University Grossman School of Medicine, New York; 3 Department of Population Health, New York University Grossman School of Medicine, New York; 4 Department of Epidemiology, Johns Hopkins Bloomberg School of Public Health, Baltimore, Maryland; 5 Department of Medical Social Sciences, Northwestern University, Chicago, Illinois; 6 Department of Epidemiology, Lifecourse Epidemiology of Adiposity & Diabetes Center, University of Colorado Anschutz Medical Campus, Aurora; 7 Department of Population and Public Health Sciences, Keck School of Medicine, University of Southern California, Los Angeles; 8 Department of Epidemiology, Dartmouth Geisel School of Medicine, Lebanon, New Hampshire; 9 Avera Health Institute, Sioux Falls, South Dakota; 10 Department of Pediatrics, University of Washington, Seattle; 11 Jack and Lucy Clark Department of Pediatrics, Icahn School of Medicine at Mount Sinai, New York, New York; 12 Department of Gynecology & Obstetrics, Emory University School of Medicine, Atlanta, Georgia; 13 Kaiser Permanente Northern California, Oakland; 14 Columbia University Irving Medical Center, New York State Psychiatric Institute, New York; 15 College of Engineering, Northeastern University, Boston, Massachusetts; 16 Department of Epidemiology & Biostatistics, College of Public Health, University of Georgia, Athens; 17 Department of Pediatrics, University of Wisconsin School of Medicine and Public Health, Madison; 18 Department of International Health, Johns Hopkins Bloomberg School of Public Health, Baltimore, Maryland; 19 Department of Medicine, Vanderbilt University Medical Center, Nashville, Tennessee; 20 Department of Psychiatry and Behavioral Sciences, University of California, Davis, Sacramento; 21 The MIND Institute, University of California, Davis, Sacramento; 22 Brown Center for the Study of Children at Risk, Brown Alpert Medical School and Women & Infants Hospital, Providence, Rhode Island; 23 Department of Pediatrics, Oregon Health and Science University School of Medicine, Portland; 24 Department of Psychiatry, University of Rochester Medical Center, Rochester, New York; 25 Department of Population Medicine, Harvard Pilgrim Health Care Institute, Boston, Massachusetts; 26 Department of Environmental Sciences and Engineering, Gillings School of Global Public Health, University of North Carolina at Chapel Hill, Chapel Hill; 27 Department of Family & Preventive Medicine, University of Utah School of Medicine, Salt Lake City; 28 Massachusetts General Hospital, Harvard Medical School, Boston; 29 Department of Preventive Medicine, University of Tennessee Health Science Center, Memphis; 30 Department of Psychological & Brain Sciences, Columbian College of Arts & Sciences, George Washington University, Washington, DC

## Abstract

**Importance:**

Few population-based studies in the US collected individual-level data from families during the COVID-19 pandemic.

**Objective:**

To examine differences in COVID-19 pandemic–related experiences in a large sociodemographically diverse sample of children and caregivers.

**Design, Setting, and Participants:**

The Environmental influences on Child Health Outcomes (ECHO) multi-cohort consortium is an ongoing study that brings together 64 individual cohorts with participants (24 757 children and 31 700 caregivers in this study) in all 50 US states and Puerto Rico. Participants who completed the ECHO COVID-19 survey between April 2020 and March 2022 were included in this cross-sectional analysis. Data were analyzed from July 2021 to September 2022.

**Main Outcomes and Measures:**

Exposures of interest were caregiver education level, child life stage (infant, preschool, middle childhood, and adolescent), and urban or rural (population <50 000) residence. Dependent variables included COVID-19 infection status and testing; disruptions to school, child care, and health care; financial hardships; and remote work. Outcomes were examined separately in logistic regression models mutually adjusted for exposures of interest and race, ethnicity, US Census division, sex, and survey administration date.

**Results:**

Analyses included 14 646 children (mean [SD] age, 7.1 [4.4] years; 7120 [49%] female) and 13 644 caregivers (mean [SD] age, 37.6 [7.2] years; 13 381 [98%] female). Caregivers were racially (3% Asian; 16% Black; 12% multiple race; 63% White) and ethnically (19% Hispanic) diverse and comparable with the US population. Less than high school education (vs master’s degree or more) was associated with more challenges accessing COVID-19 tests (adjusted odds ratio [aOR], 1.88; 95% CI, 1.06-1.58), lower odds of working remotely (aOR, 0.04; 95% CI, 0.03-0.07), and more food access concerns (aOR, 4.14; 95% CI, 3.20-5.36). Compared with other age groups, young children (age 1 to 5 years) were least likely to receive support from schools during school closures, and their caregivers were most likely to have challenges arranging childcare and concerns about work impacts. Rural caregivers were less likely to rank health concerns (aOR, 0.77; 95% CI, 0.69-0.86) and social distancing (aOR, 0.82; 95% CI, 0.73-0.91) as top stressors compared with urban caregivers.

**Conclusions:**

Findings in this cohort study of US families highlighted pandemic-related burdens faced by families with lower socioeconomic status and young children. Populations more vulnerable to public health crises should be prioritized in recovery efforts and future planning.

## Key Points

**Question:**

How did COVID-19 pandemic–related experiences in the United States vary by caregiver education level, child life stage, and urban vs rural residence?

**Findings:**

In this cohort study of United States families, lower caregiver education was associated with financial strain and reduced ability to adapt to pandemic-related demands, younger children received the least support from school, and their caregivers faced the most childcare challenges; experiences were generally similar for urban and rural families.

**Meaning:**

The findings suggest that COVID-19 experiences varied notably by caregiver education and child life stage in this population with implications for recovery efforts and future pandemic preparedness.

## Teaser

This cohort study evaluates COVID-19–pandemic experiences among sociodemographically diverse United States families.

## Introduction

Children and families across the US experienced dramatic alterations in their daily lives during the height of the COVID-19 pandemic, which, in addition to SARS-COV-2 infection, included disruptions to school, health care, and work; challenges accessing material goods; and additional financial strain. While notable social inequities in infection rates,^1^ disease severity,^2,3^ and financial strain^4^ have been observed among adults, less is known about the experiences of children and their caregivers. This is partially due to pandemic-related disruptions of large, ongoing studies. Thus, most studies characterizing pandemic experiences among US families have relied on smaller samples,^5^ with limited opportunities to describe inequities along multiple dimensions of social experience,^6^ or have used publicly available administrative data (eg, from the US Centers for Disease Control and Prevention [CDC]^1^), limiting insights into the immediate experiences of children and families. Understanding social inequities in pandemic experiences in population-based samples of children is essential for understanding how vulnerabilities may cluster together to widen inequities in health,^7^ informing recovery efforts, and supporting all families in the context of future large-scale disruptions.

We leveraged data from the National Institutes of Health (NIH)–funded Environmental influences on Child Health Outcomes (ECHO) consortium, which supports a US population–based cohort of children and their caregivers from more than 200 sites across the nation; collectively, these sites include participants in all 50 US states and Puerto Rico with notable socioeconomic, racial, ethnic, and geographic diversity.^8,9^ In April 2020, the ECHO consortium developed surveys capturing COVID-19 pandemic experiences. Drawing on data from more than 13 000 children and caregivers, we examined independent associations between caregiver education, child life stage, and urban vs rural area of residence with inequities in pandemic-related experiences.

## Methods

The ECHO Program brought together 69 ongoing and new pregnancy and pediatric cohorts to enroll and consent a proportion of these participants into the ECHO cohort, which was designed to be a large, population-based cohort of US children unified under a single research protocol. Of more than 60 000 participants across the 69 cohorts eligible to be consented into the ECHO program, 24 757 children, adolescents, and young adults (aged 1 to 21 years) from 64 component cohorts and 31 700 caregivers from 63 component cohorts were consented into the ECHO cohort and eligible for the COVID-19 surveys during the study period. Component cohorts were not generally sampled to be representative of a given target population. Different strategies were used across the cohorts to collect COVID-19 survey data, with the most common being remote collection via a web-based survey link. Local institutional review boards (IRBs) and/or the central ECHO IRB (Western IRB) reviewed all research methods and procedures. Written informed consent or parent or guardian permission was obtained along with child assent, as appropriate, for ECHO-wide cohort data collection protocol participation and for participation in specific cohorts. This study followed the Strengthening the Reporting of Observational Studies in Epidemiology (STROBE) reporting guideline.

Caregivers in the ECHO Program who completed the ECHO COVID-19 surveys (on behalf of their children or themselves) between April 2020 and March 2022 were considered for inclusion in this study. We used 2 distinct samples to describe the experiences of caregivers and children. In the child sample, data are derived from the parent report on child survey. If more than 1 survey per child was completed, we included the earliest survey. Because these surveys were less relevant for infants (aged 0 to 1 year), infants were excluded from the child sample.

The caregiver sample included adults who completed a separate survey about their own pandemic experiences. When multiple caregivers from the same household completed the adult survey, we selected the maternal report, as most caregivers identified as mothers of the study children. Participants who were pregnant at survey completion with no other children were excluded as our primary focus was on those with caregiving burdens. While most participants in the caregiver sample were caregivers of children in the child sample, these samples were mutually exclusive with respect to data sources used within each sample (eFigure 1 in Supplement 1).

### Measures

#### Sociodemographic Measures

Caregiver education, a component of family socioeconomic status, was measured categorically (less than high school, high school degree, some college, college degree, and master’s degree or higher). Child life stage was examined as infancy (0 to 1 year; included in the caregiver analysis only), early childhood (1 to 5 years), middle childhood (6 to 11 years), and adolescence and young adulthood (12 to 21 years). Address data were used to identify urban vs rural residence using the National Center for Health Statistics classification system; living in a metropolitan statistical area with fewer than 50 000 people was considered rural.^10^ Race and ethnicity data were either self-reported (for the caregivers) or caregiver-reported (for the children) and collected in accordance with NIH requirements related to reporting individual-level participant data.

#### Pandemic-Related Experiences

ECHO COVID-19 surveys are available at the NIH DR2 website.^11^ In the child sample, we examined items related to COVID-19 infection, access to COVID-19 testing, changes to health care, and disruptions to school, preschool or day care and related services.

From the caregiver survey, we examined whether caregivers transitioned to remote work, had difficulty arranging childcare, or changed their work schedule to care for children. We also examined which COVID-19–related stressors (eg, access to food or supplies, financial concerns, health concerns, social distancing and quarantining, impacts of child, family, work, and/or community) caregivers ranked as top stressors.

All survey items were coded as yes (1) if the child or caregiver had the experience or no (0). See the eMethods in Supplement 1 for additional information.

### Statistical Analysis

We describe unadjusted differences in pandemic-related experiences by 3 sociodemographic characteristics of interest: caregiver education, child life stage, and urban vs rural residence. To estimate the independent contribution of these 3 variables to inequities in pandemic experiences, we generated separate logistic regression models for each experience (the dependent variable), mutually adjusted for all 3 variables. Adjusted models also included a categorical variable capturing when surveys were completed in relation to different phases of the pandemic (April 2020 to July 2020, August 2020 to November 2020, December 2020 to March 2021, April 2021 to July 2021, and August 2021 or later);^12,13^ sex, race, and ethnicity of the child for child analyses or of the caregiver for caregiver analyses; and region of residence as defined by US Census divisions to broadly account for geographic variation in COVID-19 related policies.^14^ Race and ethnicity were conceptualized as social constructs that reflect membership in marginalized groups that have experienced inequitable burdens of COVID-19 infection and disease severity^2^ and fewer returns on educational investments with respect to health and financial gains.^15^ Odds ratios were considered statistically significant at *P* < .05.

The analytic data set included participants complete for COVID-19 survey items. Missing covariates were imputed using multivariate imputation by chained equations.^16^ A select number of outcomes were defined using skip logic and are therefore based on a subsample of participants (eMethods in Supplement 1). Analyses were conducted in R version 4.1.0 (R Foundation).

## Results

The child analytic sample included 14 646 participants (mean [SD] age, 7.1 [4.4] years; 7120 [49%] female) from 57 cohorts. A comparison of this sample with eligible children in the ECHO cohort (N = 24 757; 64 cohorts) indicated that these samples were largely similar with respect to child sex, ethnicity, race, and maternal highest level of education but included a larger proportion of rural participants (16% vs 13% in the ECHO cohort) (Table 1). The child sample was racially and ethnically diverse, with 419 children (3%) identifying as American Indian or Alaska Native, 447 (3%) as Asian, 1989 (14%) as Black, 3432 (24%) as Hispanic, and 9145 (65%) as White, and 1443 (10%) as more than 1 race.

**Table 1.** Demographic Characteristics of the Child and Caregiver Analytic Samples Compared With the Full ECHO-Wide Cohort

Characteristic	Participants, No. (%)				US adult population, %^a^
	Children		Caregivers		
	Analytic sample (N = 14 646) [57 cohorts]	ECHO-wide cohort (N = 24 757) [64 cohorts]	Analytic sample (N = 13 653) [60 cohorts]	ECHO-wide cohort (N = 31 700) [64 cohorts]	
Age, mean (SD), y	7.1 (4.4)	7.9 (5.1)	37.6 (7.2)	36.8 (7.1)	NA
Sex					
Responded	14 643 (99.0)	24 734 (99.0)	13 628 (99.0)	31 647 (99.0)	NA
Male	7523 (51.4)	12 770 (51.6)	219 (1.6)	249 (<1)	NA
Female	7120 (48.6)	11 964 (48.4)	13 381 (98.4)	30 708 (99.0)	NA
Ethnicity^b^	14 567 (99.0)	24 499 (99.0)	13 533 (99.0)	30 918 (97.5)	NA
Hispanic	3432 (23.6)	5894 (24.1)	2519 (18.6)	8089 (26.2)	18.8
Non-Hispanic	11 135 (76.4)	18 605 (75.9)	11 014 (81.4)	22 829 (73.8)	81.2
Race^b^^,^^c^	14 144 (96.6)	23 554 (95.1)	13 091 (95.9)	29 347 (92.6)	NA
American Indian or Alaska Native	419 (3.0)	693 (2.9)	340 (2.6)	855 (2.9)	1.0
Asian^d^	447 (3.2)	752 (3.2)	418 (3.2)	996 (3.4)	5.8
Black	1989 (14.1)	3807 (16.2)	2131 (16.3)	4480 (15.3)	12.1
Multiple race	1443 (10.2)	2996 (12.7)	1555 (11.9)	3603 (12.3)	12.6
White	9145 (64.7)	14 199 (60.3)	8245 (63.0)	17 782 (60.6)	61.2
Other^e^	701 (5.0)	1107 (4.7)	402 (3.1)	1631 (5.6)	7.2
Life stage at COVID-19 survey	14 646 (100)	24 757 (100)	13 496 (99.0)	31 444 (99.0)	NA
Infant	0	0	1262 (9.4)	1033 (3.3)	NA
Early childhood	6758 (46.1)	10 625 (42.9)	4690 (34.8)	13 157 (41.8)	NA
Middle childhood	6220 (42.5)	9538 (38.5)	5237 (38.8)	11 583 (36.8)	NA
Adolescence	1668 (11.4)	4594 (18.6)	2307 (17.1)	5671 (18.0)	NA
Maternal education	14 218 (97.1)	23 285 (94.1)	13 220 (96.8)	29 681 (93.6)	NA
<High school	754 (5.3)	1367 (5.9)	603 (4.6)	2026 (6.8)	11.1
High school	1767 (12.4)	3143 (13.5)	1508 (11.4)	4006 (13.5)	26.5
Some college	3795 (26.7)	6304 (27.1)	3320 (25.1)	7316 (24.6)	28.7
Bachelor’s degree	4111 (28.9)	6437 (27.6)	3875 (29.3)	8224 (27.7)	20.6
≥Master’s degree	3791 (26.7)	6034 (25.9)	3914 (29.6)	8109 (27.3)	13.1
Census division	14 606 (99.0)	14 606 (99.0)	13 606 (99.0)	31 004 (97.8)	NA
Division 1	1994 (13.7)	3688 (15.1)	2232 (16.4)	5052 (16.3)	NA
Division 2	2142 (14.7)	3377 (13.8)	1877 (13.8)	4691 (15.1)	NA
Division 3	1482 (10.2)	2658 (10.9)	1479 (10.9)	2874 (9.3)	NA
Division 4	1781 (12.2)	2426 (9.9)	2028 (14.9)	3237 (10.4)	NA
Division 5	1846 (12.6)	2596 (10.6)	1691 (12.4)	4715 (15.2)	NA
Division 6	698 (4.8)	1578 (6.5)	536 (3.9)	1585 (5.1)	NA
Division 7	780 (5.3)	1201 (4.9)	193 (1.4)	1470 (4.7)	NA
Division 8	1251 (8.6)	2156 (8.8)	979 (7.2)	2251 (7.3)	NA
Division 9	2632 (18.0)	4756 (19.5)	2591 (19.0)	5129 (16.5)	NA
Urban vs rural (NCHS)	12 973 (88.6)	18 045 (72.9)	12 622 (92.5)	20 843 (65.8)	
Urban	10 930 (84.3)	15 743 (87.2)	10 600 (84.0)	17 951 (86.1)	85.0
Rural	2043 (15.8)	2302 (12.8)	2022 (16.0)	2892 (13.9)	15.0

Abbreviations: ECHO, Environmental influences on Child Health Outcomes; NA, not applicable; NCHS, National Center for Health Statistics.

^a^ Data are derived from the American Community Survey^17^ and 2010 Decennial Census.^18^

^b^ Race and ethnicity data were collected via participant self- and parent-report and reported in accordance with National Institutes of Health requirements related to reporting of individual-level participant data.

^c^ For the race variable, participants were asked to select all that apply from the following categories: American Indian or Alaska Native, Asian Indian, Other Asian (which, if selected, expanded to include Chinese, Filipino, Japanese, Korean, Vietnamese, Other Asian, and do not know), Native Hawaiian or Other Pacific Islander, some other race, prefer not to answer, and do not know. Participants were categorized in the multiple race group if they selected more than 1 race for this question.

^d^ Due to small cell sizes and concerns about identifiability, those identifying as Asian and Other Asian were combined.

^e^ Due to small cell sizes and concerns about identifiability, individuals identifying as Native Hawaiian or Other Pacific Islander were combined with participants who selected other and did not specify.

The caregiver analytic sample of 13 653 participants (mean [SD] age, 37.6 [7.2] years; 13 381 [98%] female) from 60 cohorts was also largely similar to the broader ECHO Cohort (N = 31 700, 64 cohorts) but included relatively fewer Hispanic participants (19% vs 26%) and more rural participants (16% vs 13%) (Table 1); 13 287 caregivers (97.5%) in the sample identified as the child’s mother. A comparison to US Census data^17,18^ indicates the caregiver sample is comparable with the US population with respect to race and ethnicity but overrepresentative of Black caregivers and underrepresentative of Asian caregivers. It also underrepresents caregivers with the lowest levels of education (5% in the analytic sample have less than a high school education compared with 11% in the US). Across both samples, participants resided in all 9 Census divisions, indicating broad geographic diversity. Most surveys were completed prior to 2022 (eFigure 2 in Supplement 1) and remotely (10 838 of 14 646 [74%] for the child sample and 10 922 of 13 653 [80%] for the caregiver sample).

### Sociodemographic Differences in Child Pandemic-Related Experiences

#### Child COVID-19 Infection and Testing

During the study period, 858 children (5.9%) had a COVID-19 infection, with the odds of infection highest among children whose caregivers had some college (adjusted OR [aOR], 1.29; 95% CI, 1.05-1.60) or a college degree (aOR, 1.42; 95% CI, 1.16-1.73) compared with those with a master’s degree or higher (Table 2). However, among the 4030 caregivers who wanted to get their child tested for COVID-19, those with less than a high school education (aOR, 1.88; 95% CI, 1.06-1.58) or a high school education (aOR, 1.97; 95% CI, 1.32-2.96) were more likely to be unable to get the test compared with those with a master’s level or higher education. Compared with those in early childhood, adolescents were less likely to have problems accessing COVID-19 tests (aOR, 0.42; 95% CI, 0.25-0.69). No differences by urban or rural residence were observed.

**Table 2.** Differences in Child Health Care, Infection, and Testing by Maternal Education

Outcome	No. (%)		OR (95% CI)	
	Yes	No	Unadjusted	Adjusted^a^
**Caregiver cancelled health care appointments (n = 14 646 with data)**				
Overall	2245 (15.3)	12 401 (84.7)	NA	NA
Maternal education				
<High school	154 (20.0)	615 (80.0)	1.46 (1.19-1.78)	1.27 (1.02-1.58)
High school	326 (17.9)	1496 (82.1)	1.28 (1.10-1.48)	1.20 (1.02-1.41)
Some college	657 (16.7)	3267 (83.3)	1.17 (1.04-1.33)	1.15 (1.01-1.32)
Bachelor’s degree	541 (12.8)	3700 (87.2)	0.85 (0.75-0.97)	0.87 (0.77-1.00)
≥Master’s degree	567 (14.6)	3323 (85.4)	1 [Reference]	1 [Reference]
**Health care professional cancelled appointments (n = 14 646 with data)**				
Overall	2342 (16.0)	12 304 (84.0)	NA	NA
Maternal education				
Less than high school	158 (20.6)	611 (79.5)	1.34 (1.10-1.64)	1.26 (1.02-1.56)
High school	288 (15.8)	1534 (84.2)	0.99 (0.85-1.17)	0.99 (0.84-1.18)
Some college	624 (15.9)	3300 (84.1)	0.99 (0.87-1.12)	1.03 (0.90-1.17)
Bachelor’s degree	648 (15.3)	3593 (84.7)	0.95 (0.84-1.07)	0.96 (0.85-1.09)
≥Master’s degree	624 (16.0)	3266 (84.0)	1 [Reference]	1 [Reference]
**Child tested positive for COVID-19 (n = 14 646 with data)**				
Overall	858 (5.9)	13 788 (94.1)	NA	NA
Maternal education				
Less than high school	46 (6.0)	723 (94.0)	1.30 (0.93-1.81)	0.99 (0.69-1.43)
High school	92 (5.2)	1730 (95.0)	1.09 (0.83-1.42)	0.97 (0.73-1.29)
Some college	263 (6.7)	3661 (93.3)	1.44 (1.18-1.75)	1.29 (1.05-1.60)
Bachelor’s degree	274 (6.5)	3967 (93.5)	1.40 (1.15-1.71)	1.42 (1.16-1.73)
≥Master’s degree	183 (4.7)	3707 (95.3)	1 [Reference]	1 [Reference]
**Caregiver was unable to get the child tested for COVID-19 (n = 4030 with data)^b^**				
Overall	375 (9.3)	3655 (90.7)	NA	NA
Maternal education				
Less than high school	23 (12.5)	161 (87.5)	1.93 (1.17-3.19)	1.88 (1.06-3.33)
High school	68 (15.2)	379 (84.8)	2.35 (1.67-3.31)	1.97 (1.32-2.96)
Some college	104 (9.9)	947 (90.1)	1.45 (1.07-1.96)	1.33 (0.94-1.87)
Bachelor’s degree	97 (8.4)	1065 (91.7)	1.19 (0.88-1.63)	1.19 (0.86-1.65)
≥Master’s degree	83 (7.0)	1103 (93.0)	1 [Reference]	1 [Reference]

Abbreviations: NA, not applicable; OR, odds ratio.

^a^ Models were mutually adjusted for maternal education, urban or rural residence, and child life stage. Covariates included race and ethnicity (as defined in Table 1); when surveys were completed in relation to different phases of the pandemic (April 2020 to July 2020, August 2020 to November 2020, December 2020 to March 2021, April 2021 to July 2021, and August 2021 or later); and US Census division.

^b^ Sample size derived from those that answered yes to wanting to get their child tested but not being able to get their child tested (see eMethods in Supplement 1 for more details).

#### Children’s Health Care

A total of 2245 caregivers (15%) cancelled health care appointments due to COVID-19–related concerns; these cancellations were more likely among caregivers with lower levels of education and young children. Compared with those with a master’s degree, caregivers with less than a high school education had 1.27 times (95% CI, 1.02-1.58) higher odds of cancelling appointments. Compared with caregivers with children in early childhood, caregivers with children in middle childhood (aOR, 0.85; 95% CI, 0.76-0.94) and adolescence (aOR, 0.50; 95% CI, 0.42-0.60) were less likely to cancel appointments (eTable 1 in Supplement 1). A further 2342 children (16%) had health care visits cancelled by a health care professional, and these cancellations were more likely among caregivers with less than a high school education (aOR, 1.26; 95% CI, 1.02-1.56) compared with those with a master’s degree or more. No differences by urban or rural residence were observed.

#### Schooling and School-Related Supports

There were 11 878 children (81%) enrolled in school, preschool, or daycare at the time of the survey administration. Of those whose schools closed, most experienced school or daycare closure (9788 [82%]) and remote schooling (8038 [84%]) during the study period; however, closures were most common among children in middle childhood (aOR, 4.10; 95% CI, 3.64-4.62) followed by adolescents (aOR, 2.34; 95% CI, 1.99-2.75), compared with those in early childhood. As expected, almost all children older than 5 years were offered online learning (5174 [97%] of those in middle childhood and 1214 [97%] of those in adolescence); however, 1650 young children (56%) were also offered some kind of online learning support (Table 3). Overall, schools offered free internet or a computer to 2248 children (29%) and 5549 (70%), respectively. While older children were more likely to receive these supports, a large proportion of young children were offered these supports as well (340 [22%] were offered free internet, 658 [43%] a computer).

**Table 3.** Differences in Pandemic-Related School Experiences by Child Life Stage

Outcome	No. (%)		OR (95% CI)	
	Yes	No	Unadjusted	Adjusted^a^
**School closed because of the COVID-19 outbreak (n = 11 878 with data)^b^**				
Overall	9788 (82.4)	2090 (17.6)	NA	NA
Child life stage				
Early childhood	3135 (72.5)	1187 (27.5)	1 [Reference]	1 [Reference]
Middle childhood	5386 (89.2)	654 (10.8)	3.11 (2.80-3.46)	4.10 (3.64-4.62)
Adolescence	1267 (83.6)	249 (16.4)	1.92 (1.65-2.24)	2.34 (1.99-2.75)
**Child received school meals prepandemic (n = 9582 with data)^c^**				
Overall	4016 (41.9)	5566 (58.1)	NA	NA
Child life stage				
Early childhood	1318 (44.6)	1636 (55.4)	1 [Reference]	1 [Reference]
Middle childhood	2183 (40.7)	3182 (59.3)	0.85 (0.77-0.93)	0.91 (0.81-1.02)
Adolescence	515 (40.8)	748 (59.2)	0.85 (0.74-0.97)	0.66 (0.56-0.77)
**School continued to offer free meals during school closure (n = 3998 with data)^d^**				
Overall	3162 (79.1)	836 (20.9)	NA	NA
Child life stage				
Early childhood	737 (56.3)	572 (43.7)	1 [Reference]	1 [Reference]
Middle childhood	1971 (90.6)	205 (9.4)	7.46 (6.22-8.93)	7.74 (6.34-9.44)
Adolescence	454 (88.5)	59 (11.5)	5.97 (4.45-8.00)	6.83 (5.00-9.33)
**Child was able to get free meals if offered (n = 3137 with data)^e^**				
Overall	2279 (72.7)	858 (27.4)	NA	NA
Child life stage				
Early childhood	546 (74.8)	184 (25.2)	1 [Reference]	1 [Reference]
Middle childhood	1425 (73.0)	528 (27.0)	0.90 (0.74-1.10)	0.83 (0.67-1.03)
Adolescence	308 (67.8)	146 (32.2)	0.71 (0.54-0.92)	0.69 (0.52-0.91)
**School offered online learning (n = 9525 with data)** ^f^				
Overall	8038 (84.4)	1487 (15.6)	NA	NA
Child life stage				
Early childhood	1650 (56.45)	1278 (43.7)	1 [Reference]	1 [Reference]
Middle childhood	5174 (96.9)	165 (3.1)	24.2 (20.4-28.8)	35.5 (29.1-43.4)
Adolescence	1214 (96.5)	44 (3.5)	21.3 (15.6-29.1)	28.1 (20.4-38.6)
**School offered free internet (n = 7727 with data)** ^g^				
Overall	2248 (29.1)	5479 (70.9)	NA	NA
Child life stage				
Early childhood	340 (22.0)	1205 (78.0)	1 [Reference]	1 [Reference]
Middle childhood	1540 (30.9)	3450 (69.1)	1.58 (1.38-1.80)	1.97 (1.70-2.29)
Adolescence	368 (30.9)	824 (69.1)	1.58 (1.33-1.87)	1.74 (1.44-2.10)
**School offered a free computer or tablet (n = 7767 with data)** ^h^				
Overall	5549 (71.4)	2218 (28.6)	NA	NA
Child life stage				
Early childhood	658 (42.5)	890 (57.5)	1 [Reference]	1 [Reference]
Middle childhood	3949 (78.7)	1071 (21.3)	4.98 (4.41-5.63)	6.70 (5.81-7.72)
Adolescence	942 (78.6)	257 (21.4)	4.95 (4.17-5.88)	5.01 (4.15-6.04)

Abbreviations: NA, not applicable; OR, odds ratio.

^a^ Models were mutually adjusted for maternal education, urban or rural residence, and child life stage. Covariates included race and ethnicity (as defined in Table 1); when surveys were completed in relation to different phases of the pandemic (April 2020 to July 2020, August 2020 to November 2020, December 2020 to March 2021, April 2021 to July 2021, and August 2021 or later); and US Census division.

^b^ Sample excluded those who were not enrolled in school, preschool, or daycare.

^c^ In a subsample of 9788 children whose caregivers responded yes, their child’s school closed (206 missing).

^d^ In a subsample of 4016 children whose caregivers responded yes, their child received school meals prepandemic (48 missing).

^e^ In a subsample of 3163 children whose caregivers responded yes, their child’s school continued to offer meals during school closure (25 missing).

^f^ In a subsample of 9788 children whose caregivers responded yes, their child’s school closed (263 missing).

^g^ In a subsample of 9788 children whose caregivers responded yes, their child’s school closed (311 missing).

^h^ In a subsample of 9788 children whose caregivers responded yes, their child’s school closed (271 missing).

Of 3998 children who were receiving school meals at the beginning of the pandemic, 3162 (79%) had that meal service continue. However, the continuation of that service was more likely among those in middle childhood (aOR, 7.64; 95% CI, 6.34-9.44) and adolescence (aOR, 6.83; 95% CI, 5.00-9.33) compared with those in early childhood. While 2279 children (73%) who were offered free meals were able to get the meals offered, adolescents were less able to access meals compared with those in early childhood (aOR, 0.69; 95% CI, 0.52-0.91).

We observed no differences in school, preschool, or daycare closure by caregiver education or urban vs rural residence; however, children of caregivers with lower levels of education were more likely to have free meal service continue during the pandemic and also more likely to receive free home internet or a computer to support for remote learning (eTable 2 in Supplement 1). We did not observe differences in school provided supports by urban and rural residence, but it was easier for children in rural areas (aOR, 1.71; 95% CI, 1.27-2.32) to access school meals if they were provided (eTable 2 in Supplement 1).

### Sociodemographic Differences in Caregiver Pandemic-Related Experiences

#### Work and Child Care

While 4221 caregivers (31%) reported working remotely (Table 4), this proportion varied exponentially with caregiver education. The lowest rates were observed among those with less than a high school education (27 [4%]; aOR, 0.04; 95% CI, 0.03-0.07) and a high school education (144 [9%]; aOR, 0.10; 95% CI, 0.08-0.11) compared with those with a master’s degree (2085 [52%]). Even those with a bachelor’s degree were much less likely to work remotely (1370 [34%]; aOR, 0.48; 95% CI, 0.43-0.52). Similarly, caregivers with lower levels of education were less likely to change their work schedules to care for children themselves (Table 4). The opposite pattern was observed for difficulties in arranging childcare; while 2472 (18%) reported these challenges overall, 88 caregivers (14%) with less than a high school education reported these challenges compared with 871 (22%) with a master’s degree or above (aOR, 0.44; 95% CI, 0.34-0.57).

**Table 4.** Differences in Caregiver Work and Childcare Experiences by Maternal Education and Child Life Stage (n = 13 655 With Data)

Outcome	No. (%)		OR (95% CI)	
	Yes	No	Unadjusted	Adjusted^a^
**Caregiver moved to working remotely**				
Overall	4221 (30.9)	9434 (69.1)	NA	NA
Maternal education				
<High school	27 (4.4)	594 (95.7)	0.04 (0.03-0.07)	0.05 (0.03-0.07)
High school	144 (9.2)	1424 (90.8)	0.10 (0.08-0.11)	0.10 (0.08-0.12)
Some college	595 (17.2)	2864 (82.8)	0.19 (0.17-0.21)	0.19 (0.17-0.22)
Bachelor’s degree	1370 (34.3)	2624 (65.7)	0.48 (0.43-0.52)	0.48 (0.44-0.53)
≥Master’s degree	2085 (52.0)	1928 (48.0)	1 [Reference]	1 [Reference]
Child life stage				
Infant	310 (24.2)	969 (75.8)	0.70 (0.60-0.80)	0.76 (0.65-0.90)
Early childhood	1486 (31.3)	3257 (68.7)	1 [Reference]	1 [Reference]
Middle childhood	1707 (32.2)	3589 (67.8)	1.04 (0.95-1.13)	0.98 (0.89-1.09)
Adolescence	718 (30.7)	1619 (69.3)	0.97 (0.87-1.08)	0.88 (0.78-1.00)
**Difficulty arranging childcare**				
Overall	2472 (18.1)	11 183 (81.9)	NA	NA
Maternal education level				
<High school	88 (14.2)	533 (85.8)	0.58 (0.46-0.74)	0.44 (0.34-0.57)
High school	273 (17.4)	1296 (82.6)	0.74 (0.63-0.87)	0.57 (0.48-0.69)
Some college	598 (17.3)	2850 (82.7)	0.76 (0.67-0.86)	0.67 (0.58-0.77)
Bachelor’s degree	642 (16.1)	3359 (84.0)	0.68 (0.61-0.76)	0.69 (0.61-0.78)
≥Master’s degree	871 (21.7)	3145 (78.3)	1 [Reference]	1 [Reference]
Child life stage				
Infant	204 (15.9)	1076 (84.1)	0.55 (0.46-0.64)	0.62 (0.52-0.73)
Early childhood	1209 (25.5)	3534 (74.5)	1 [Reference]	1 [Reference]
Middle childhood	949 (17.9)	4346 (82.1)	0.63 (0.57-0.70)	0.69 (0.62-0.77)
Adolescence	110 (4.7)	2227 (95.3)	0.14 (0.11-0.17)	0.14 (0.11-0.17)
**Changed work schedule to care for children**				
Overall	3364 (24.6)	10 291 (75.4)	NA	NA
Maternal education level				
<High school	71 (11.4)	552 (88.6)	0.21 (0.16-0.27)	0.23 (0.17-0.30)
High school	186 (11.9)	1383 (88.2)	0.21 (0.18-0.26)	0.23 (0.19-0.28)
Some college	547 (15.9)	2890 (84.1)	0.30 (0.27-0.33)	0.32 (0.28-0.36)
Bachelor’s degree	1013 (25.3)	2995 (74.7)	0.54 (0.49-0.59)	0.55 (0.50-0.61)
≥Master’s degree	1547 (38.5)	2471 (61.5)	1 [Reference]	1 [Reference]
Child life stage				
Infant	243 (19.0)	1036 (81.0)	0.49 (0.42-0.57)	0.58 (0.49-0.68)
Early childhood	1526 (32.2)	3219 (67.8)	1 [Reference]	1 [Reference]
Middle childhood	1442 (27.2)	3852 (72.8)	0.78 (0.72-0.85)	0.80 (0.72-0.88)
Adolescence	153 (6.6)	2184 (93.5)	0.14 (0.12-0.17)	0.12 (0.10-0.14)

Abbreviations: NA, not applicable; OR, odds ratio.

^a^ Models were mutually adjusted for maternal education, urban or rural residence, and child life stage. Covariates included race and ethnicity (as defined in Table 1); when surveys were completed in relation to different phases of the pandemic (April 2020 to July 2020, August 2020 to November 2020, December 2020 to March 2021, April 2021 to July 2021, and August 2021 or later); and US Census division.

Caregivers in rural areas were also less likely to work remotely than those in urban areas (aOR, 0.78; 95% CI, 0.69-0.89) as were caregivers with infant children compared with those with children in early childhood (aOR, 0.76; 95% CI, 0.65-0.90) (eTable 3 in Supplement 1). Caregivers with infants (aOR, 0.62; 95% CI, 0.53-0.73), children in middle childhood (aOR, 0.69; 95% CI, 0.62-0.77), and adolescents (aOR, 0.14; 95% CI, 0.11-0.17) had less difficulty arranging childcare than those with children in early childhood; caregivers with children in early childhood were also most likely to change their work schedules to care for children.

#### Pandemic-Related Stressors

Overall, most caregivers selected the pandemic’s impact on their child (8467 [62%]) and social distancing (7082 [52%]) as the greatest sources of pandemic stress (Table 5); however, we observed notable, dose-response associations between the stressors endorsed and caregiver education. For example, 178 caregivers (28%) with less than a high school education ranked access to food as a top stressor compared with 223 (6%) with a master’s degree and above (aOR, 4.14; 95% CI, 3.20-5.36). Similar patterns were observed for access to supplies and financial concerns. In contrast, 2373 caregivers (59%) with a master’s degree ranked social distancing as a top stressor compared with 267 (43%) with less than a high school education (aOR, 0.56; 95% CI, 0.46-0.67). Similar patterns were observed for health concerns and impacts on work, children, or the community.

**Table 5.** Differences in Top Pandemic-Related Stressors by Caregiver Education (n = 13 655 With Data)

Outcome characteristic	No. (%)		OR (95% CI)	
	Yes	No	Unadjusted	Adjusted^a^
**Access to food**				
Overall	1641 (12.0)	12 014 (88.0)	NA	NA
Maternal education				
<High school	178 (28.4)	449 (71.6)	6.59 (5.21-8.33)	4.14 (3.20-5.36)
High school	309 (19.8)	1253 (80.2)	4.26 (3.54-5.13)	3.05 (2.50-3.73)
Some college	577 (16.8)	2863 (83.2)	3.43 (2.89-4.08)	2.72 (2.25-3.28)
Bachelor’s degree	354 (8.8)	3656 (91.2)	1.65 (1.38-1.97)	1.57 (1.31-1.88)
≥Master’s degree	223 (5.6)	3793 (94.5)	1 [Reference]	1 [Reference]
**Access to supplies**				
Overall	3577 (26.2)	10 078 (73.8)	NA	NA
Maternal education				
<High school	237 (38.1)	385 (61.9)	2.56 (2.13-3.08)	2.06 (1.69-2.51)
High school	544 (34.7)	1025 (65.3)	2.28 (1.99-2.60)	1.95 (1.69-2.26)
Some college	1122 (32.6)	2321 (67.4)	2.05 (1.83-2.29)	1.85 (1.64-2.09)
Bachelor’s degree	908 (22.7)	3095 (77.3)	1.26 (1.12-1.41)	1.23 (1.10-1.38)
≥Master’s degree	766 (19.1)	3252 (80.9)	1 [Reference]	1 [Reference]
**Financial concerns**				
Overall	4697 (34.4)	8958 (65.6)	NA	NA
Maternal education				
<High school	303 (48.6)	321 (51.4)	3.03 (2.54-3.61)	2.02 (1.67-2.44)
High school	739 (47.1)	829 (52.9)	2.91 (2.57-3.30)	2.20 (1.92-2.52)
Some college	1516 (44.1)	1924 (55.9)	2.55 (2.31-2.82)	2.14 (1.92-2.38)
Bachelor’s degree	1190 (29.6)	2825 (70.4)	1.37 (1.24-1.52)	1.33 (1.20-1.47)
≥Master’s degree	949 (23.7)	3059 (76.3)	1 [Reference]	1 [Reference]
**Health concerns**				
Overall	6027 (44.1)	7628 (55.9)	NA	NA
Maternal education				
<High school	274 (44.1)	347 (55.9)	0.87 (0.73-1.04)	0.71 (0.59-0.86)
High school	601 (38.3)	968 (61.7)	0.69 (0.61-0.78)	0.62 (0.54-0.71)
Some college	1494 (43.4)	1948 (56.6)	0.85 (0.77-0.93)	0.80 (0.73-0.89)
Bachelor’s degree	1762 (44.0)	2245 (56.0)	0.86 (0.79-0.94)	0.87 (0.80-0.95)
≥Master’s degree	1896 (47.2)	2120 (52.8)	1 [Reference]	1 [Reference]
**Social distancing/quarantine**				
Overall	7082 (51.9)	6573 (78.1)	NA	NA
Maternal education				
<High school	267 (42.7)	358 (57.3)	0.51 (0.43-0.60)	0.56 (0.46-0.67)
High school	622 (39.7)	946 (60.3)	0.45 (0.40-0.51)	0.50 (0.44-0.57)
Some college	1604 (46.6)	1836 (53.4)	0.59 (0.53-0.65)	0.63 (0.57-0.70)
Bachelor’s degree	2216 (55.3)	1795 (44.8)	0.84 (0.77-0.92)	0.87 (0.79-0.95)
≥Master’s degree	2373 (59.2)	1638 (40.8)	1 [Reference]	1 [Reference]
**Impact on child**				
Overall	8467 (62.0)	5188 (38.0)	NA	NA
Maternal education				
<High school	289 (46.5)	332 (53.5)	0.36 (0.30-0.43)	0.42 (0.34-0.50)
High school	753 (47.9)	818 (52.1)	0.39 (0.34-0.44)	0.44 (0.39-0.51)
Some college	2025 (58.8)	1418 (41.2)	0.60 (0.55-0.67)	0.67 (0.61-0.75)
Bachelor’s degree	2587 (64.6)	1416 (35.4)	0.77 (0.70-0.85)	0.82 (0.74-0.90)
≥Master’s degree	2813 (70.0)	1204 (30.0)	1 [Reference]	1 [Reference]
**Impact on community**				
Overall	4430 (32.4)	9225 (67.6)	NA	NA
Maternal education				
<High school	171 (27.5)	452 (72.6)	0.61 (0.50-0.74)	0.65 (0.53-0.79)
High school	369 (23.5)	1199 (76.5)	0.50 (0.44-0.57)	0.54 (0.47-0.63)
Some college	980 (28.4)	2468 (71.6)	0.65 (0.59-0.72)	0.68 (0.61-0.76)
Bachelor’s degree	1395 (34.9)	2604 (65.1)	0.88 (0.80-0.96)	0.89 (0.81-0.98)
≥Master’s degree	1515 (37.7)	2502 (62.3)	1 [Reference]	1 [Reference]
**Impact on work**				
Overall	4821 (35.3)	8834 (64.7)	NA	NA
Maternal education				
<High school	203 (32.6)	420 (67.4)	0.66 (0.55-0.79)	0.56 (0.46-0.67)
High school	481 (30.7)	1085 (69.3)	0.59 (0.52-0.67)	0.54 (0.47-0.62)
Some college	1128 (32.8)	2314 (67.2)	0.66 (0.60-0.72)	0.62 (0.56-0.69)
Bachelor’s degree	1315 (32.8)	2695 (67.2)	0.66 (0.60-0.72)	0.65 (0.59-0.72)
≥Master’s degree	1694 (42.2)	2320 (57.8)	1 [Reference]	1 [Reference]

Abbreviations: NA, not applicable; OR, odds ratio.

^a^ Models were mutually adjusted for maternal education, urban or rural residence, and child life stage. Covariates included race and ethnicity (as defined in Table 1); when surveys were completed in relation to different phases of the pandemic (April 2020 to July 2020, August 2020 to November 2020, December 2020 to March 2021, April 2021 to July 2021, and August 2021 or later); and US Census division.

Top stressors also varied by child life stage. Concerns about access to supplies were greater for caregivers when children were younger (eTable 4 in Supplement 1). For example, 527 caregivers (22%) with adolescents were concerned about supplies compared with 397 (31%) with infants. Caregivers with children in middle childhood were most likely to rank impact on child as a top stressor (aOR, 1.21; 95% CI, 1.10-1.32, with early childhood as reference). Caregivers with adolescents were least likely to endorse impact on work (676 [28%] vs 1828 [36%] in early childhood; aOR, 0.67; 95% CI, 0.60-0.75) and social distancing (1079 [46%] vs 2586 [54%] in early childhood; aOR, 0.72; 95% CI, 0.65-0.81) as a top stressor. Compared with caregivers in urban areas, caregivers in a rural setting were less likely to endorse health concerns (aOR, 0.77; 95% CI, 0.69-0.86), social distancing (aOR, 0.82; 95% CI 0.73-0.91), and impact on the community (aOR, 0.78; 95% CI, 0.69-0.89) among their top stressors (eTable 4 in Supplement 1).

## Discussion

Leveraging data from the NIH ECHO cohort, we examined sociodemographic differences in pandemic-related experiences in more than 13 000 children and caregivers, making this the largest US study of its kind to our knowledge. Consistent with prior literature, caregiver education was associated with multiple child and caregiver pandemic-related experiences.^19,20,21^ We found that caregivers with lower levels of education were more likely to cancel health care appointments for their children due to COVID-19 related concerns, which extends work in adults to children^22^ and highlights the potential role of caregiver beliefs and attitudes in explaining socioeconomic disparities in child health care during the pandemic.^23^ Consistent with prior geospatial analyses and household survey data,^24,25,26^ caregivers with lower education were much less likely to be able to work remotely or change work schedules to care for their children, indicating that pandemic-related flexibility at work was largely experienced by those with the highest levels of education. Similarly, caregivers with lower levels of education were more likely to endorse finances and accessing food and supplies as top stressors, which is consistent with evidence suggesting that pandemic-related financial insecurity was the most salient issue facing many families with lower socioeconomic status.^27^ In contrast, caregivers with higher levels of education were more likely to endorse social distancing and quarantine or impacts on work or their child as top stressors.

Consistent with geospatial evidence suggesting greater rates of child care closures in areas with higher levels of education,^28^ Caregivers with lower levels of education were less likely to have difficulty arranging child care. Informal child care arrangements more often used among families with lower socioeconomic status^29^ may have been less impacted by care center closures, providing an important source of relative resilience to child care disruptions in a population hard hit by pandemic stressors. Collectively our findings suggest that one’s ability to respond to and cope with changing pandemic-related demands and disruptions, particularly with respect to financial stressors, work flexibility, and child care, were strongly patterned by caregiver education and highlight the sometimes extreme socioeconomic heterogeneity in the impact of the pandemic on children and families.

We also observed several differences in pandemic-related experiences by child life stage. Consistent with national data,^30^ remote learning and supports to facilitate remote learning were more common for older children, with children aged 1 to 5 years being the least likely to receive these supports. Not surprisingly, caregivers of young children also reported the most difficultly arranging childcare (including in comparison to caregivers with infants), were the most likely to need to change their work schedules to care for children and to report impact on work as a top pandemic-related stressor. Similarly, while many children aged 1 to 5 years were receiving school meals at the beginning of the pandemic, these children were the least likely to see this service continue throughout the pandemic. Collectively, findings indicate that young children and their caregivers received the fewest continuing supports from childcare and learning environments. This is consistent with studies in the US^5,31^ and in other countries^32^ indicating that young children were the hardest hit by the pandemic with respect to socioemotional development and learning^33^ and that caregivers of these children experienced significant stressors and worsening mental health during the pandemic.

While urban-rural differences in COVID-19 infection have been documented,^34^ no national studies have examined differences in other pandemic-related experiences. We observed few differences in COVID-19–related experiences by urban or rural residence. Rural caregivers were less likely to work remotely or to report being stressed about social distancing or COVID-19–related health concerns, somewhat in contrast to a greater COVID-19 mortality rate experienced in rural areas.^35,36^ Children in rural areas were more able to access school-provided meals and less likely to have disruptions in health care. Collectively, findings suggest that rural caregivers may have experienced somewhat less COVID-19 related stress and disruption to their work lives compared urban caregivers. Though US data are limited, findings are consistent with work done in the early phases of the pandemic in China.^37^

The infection rate among children in our sample was low (5.9%),^38^ reflecting that most surveys were completed prior to November 2021 before the wide spread of the more contagious Omicron variant and when school and child care closings may have limited community spread (see eFigure 2 in Supplement 1 for the distribution of surveys over time). Contrary to prior studies,^4^ we observed that COVID-19 infection was more common in children with higher socioeconomic status. However, while testing access was limited for everyone during much of the study period, our results indicate that caregivers with lower education levels had a harder time procuring tests. Thus, the lower rate of infection among families with lower socioeconomic status in our sample may be an artifact of socioeconomic inequities in test availability. Though US data on testing disparities is limited,^4^ this is a pattern consistent with what was observed in New York City early in the pandemic^39^ and in other studies of disparities in testing.^40^

### Limitations

The generalizability of ECHO data are limited in that cohorts were not sampled to be representative of specific target populations. While our study sample largely reflects US population characteristics with respect to race, our study underrepresents caregivers with lower levels of education and Hispanic ethnicity. The few population-based studies of children that continued data collection during the pandemic faced similar challenges where families with higher resources were more likely to provide data.^41^ Even representative panel-based studies faced challenges during the COVID-19 pandemic, reporting completion rates as low as 50%.^5^ Thus, results from these studies likely do not capture the experiences of those in the most adverse circumstances who may be most impacted by pandemic-related hardships and also less able to respond to midpandemic surveys. Our infection rates, based on self-report data, are limited by testing availability and inequities in testing access during the study period.

## Conclusions

In this cohort study of US families, we examined socioeconomic, child life stage, and urban-rural differences in pandemic-related experiences in one of the few studies with population-based, individual-level data on children and families. These findings highlighted the additional pandemic-related burdens faced by families with lower socioeconomic status and young children, and the need to prioritize future supports and recovery efforts to subgroups particularly vulnerable to public health crises. Results point to future investigation of more nuanced associations between the outcomes examined here as well as the long-term impacts of COVID-19 infection and pandemic-related adversities as children in the ECHO cohort continue to grow.

## Notes

Supplement 1. **eFigure 1.** Participant Flow Diagram. (A) Child Sample Flow Diagram. (B) Caregiver Sample Flow Diagram
eMethods
**eFigure 2.** Distribution of COVID-19 surveys over time (A) Child Sample (B) Caregiver Sample **eTable 1.** Differences in child health care, infection, and testing by all exposures of interest **eTable 2.** Differences in pandemic-related school experiences by all exposures of interest **eTable 3.** Differences in caregiver work and childcare experiences by all exposures of interest **eTable 4.** Differences in top pandemic-related stressors by all exposures of interest

Supplement 2.
Nonauthor Collaborators

Supplement 3.
Data Sharing Statement
