# Supplementary material for: Sociodemographic Differences in COVID-19 Pandemic Experiences Among Families in the United States
Source: JAMA Netw Open. 2023 Aug 23;6(8):e2330495. doi: 10.1001/jamanetworkopen.2023.30495 (PMC10448300; doi:10.1001/jamanetworkopen.2023.30495)
Supplement: Supplement 1. — eFigure 1. Participant Flow Diagram. (A) Child Sample Flow Diagram. (B) Caregiver Sample Flow Diagram eMethods eFigure 2. Distribution of COVID-19 surveys over time (A) Child Sample (B) Caregiver Sample eTable 1. Differences in child health care, infection, and testing by all exposures of interest eTable 2. Differences in pandemic-related school experiences by all exposures of interest eTable 3. Differences in caregiver work and childcare experiences by all exposures of interest eTable 4. Differences in top pandemic-related stressors by all exposures of interest [file jamanetwopen-e2330495-s001.pdf]

Supplemental Online Content

LeWinn KZ, Trasande L, Law A, et al; the Environmental Influences on Child Health Outcomes consortium. Sociodemographic differences in COVID-19 pandemic experiences among families in the United States. *JAMA Netw Open*. 2023;6(8):e2330495. doi:10.1001/jamanetworkopen.2023.30495

eFigure 1. Participant Flow Diagram. (A) Child Sample Flow Diagram. (B) Caregiver Sample Flow Diagram

eMethods

eFigure 2. Distribution of COVID-19 surveys over time (A) Child Sample (B) Caregiver Sample

eTable 1. Differences in child health care, infection, and testing by all exposures of interest

eTable 2. Differences in pandemic-related school experiences by all exposures of interest

eTable 3. Differences in caregiver work and childcare experiences by all exposures of interest

eTable 4. Differences in top pandemic-related stressors by all exposures of interest

This supplemental material has been provided by the authors to give readers additional information about their work.

© 2023 LeWinn KZ et al. *JAMA Network Open.*

eFigure 1. Participant Flow Diagram. (A) Child Sample Flow Diagram. (B) Caregiver Sample Flow Diagram.

(A)

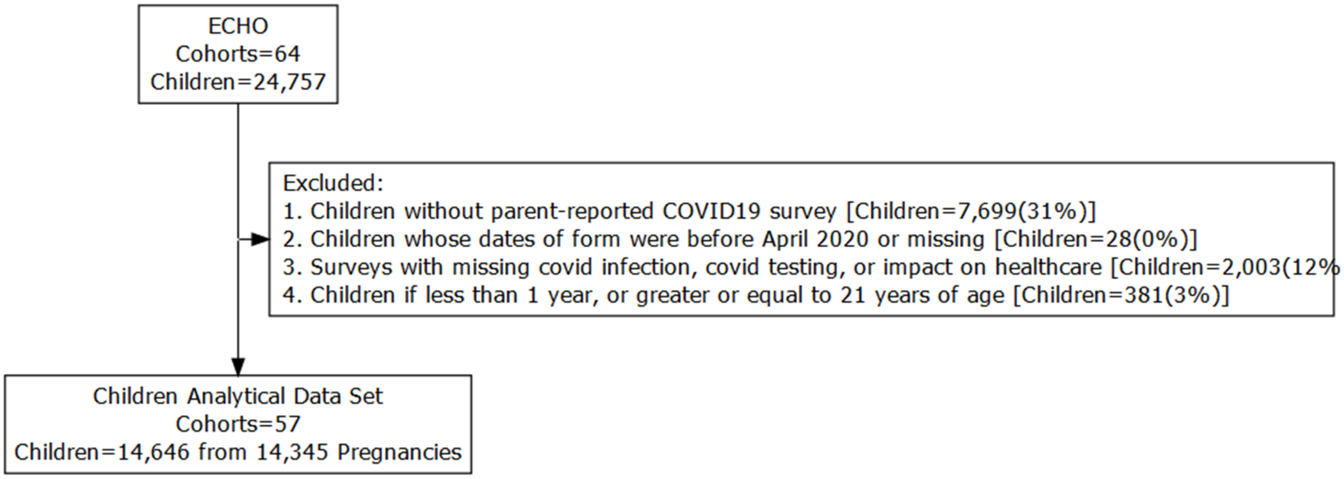

(B)

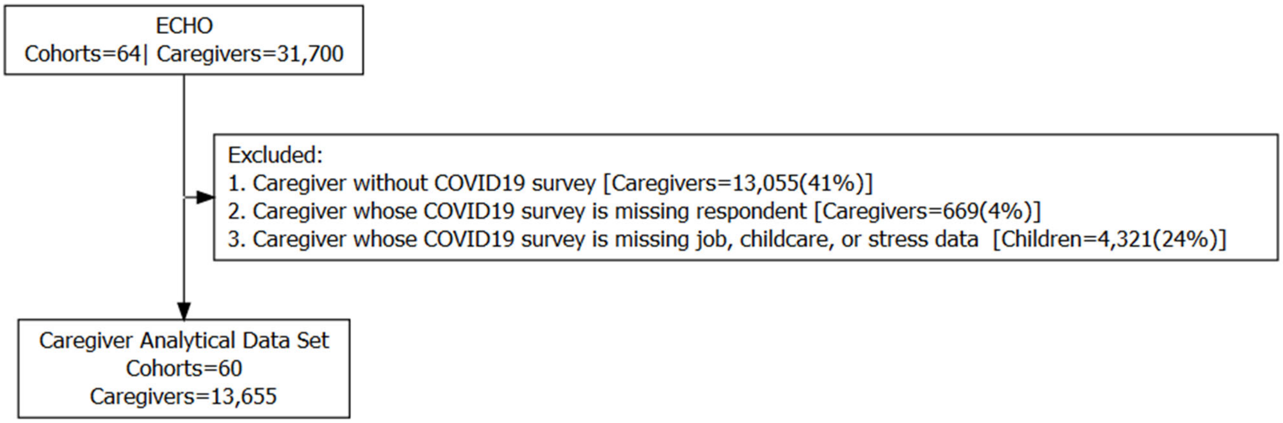

© 2023 LeWinn KZ et al. *JAMA Network Open.*

eMethods

Description of Survey Items Used in the Current Study

The full COVID-19 surveys developed for the Environmental influences on Child Health Outcomes (ECHO) Consortium can be found here:

https://tools.niehs.nih.gov/dr2/index.cfm/resource/21805

In the current study, we examined select experiences understudied in population-based contexts or in relation to children and families in the U.S. All possible choices for each response option were analyzed descriptively prior to analyses. Selections that received relatively few endorsements were also excluded from the study. We limited the scope of our paper to focus on items that reflect objective experiences (rather than behaviors or coping strategies) and items relevant to understanding social inequalities in COVID-19 experiences.

Child Sample Survey Items

Items were derived from the Parent-Report Primary Version of the ECHO COVID-19 surveys. All items were reported by caregivers about their child.

Children were included in the child sample if their caregivers provided responses for items that did not use any skip logic (COVID-19 infection, disruptions to health care, and all caregivers that responded to question #1 of the school based items).

COVID-19 infection

Question: Has a healthcare provider ever told you that the child has, or likely has, COVID-19 (Coronavirus)?

- 01 Yes
- 02 No

CODING: Yes responses to this survey items were coded as “1” and No responses as “0.”

Ability to procure a COVID-19 test

This outcome was derived by combining responses to the following three survey items.

© 2023 LeWinn KZ et al. *JAMA Network Open.*

3. Has the child had the nose swab test for the virus that causes COVID-19? (***Mark all that apply***)

- ☐01 No, I never tried to get the child tested
- ☐02 No, I tried to get the child tested but was not able to
- ☐03 Yes, and the child is waiting for the results
  - ***If yes*** ➔ **3.a.** When was the date of the child's most recent test? __ __/__ __ __ __ *mm yyyy*
- ☐04 Yes, and the test showed that the child does not have it ("**negative**" test)
  - ***If yes*** ➔ **3.b.** When was the date of the child's most recent **negative** test? __ __/__ __ __ __ *mm yyyy*
- ☐05 Yes, and the test showed that the child does have it ("**positive**" test)
  - ***If yes*** ➔ **3.c.** When was the date of the child's most recent **positive** test? __ __/__ __ __ __ *mm yyyy*

4. Has the child had the mouth swab test for the virus that causes COVID-19? (***Mark all that apply***)

- ☐01 No, I never tried to get the child tested
- ☐02 No, I tried to get the child tested but was not able to
- ☐03 Yes, and the child is waiting for the results
  - ***If yes*** ➔ **4.a.** When was the date of the child's most recent test? __ __/__ __ __ __ *mm yyyy*
- ☐04 Yes, and the test showed that the child does not have it ("**negative**" test)
  - ***If yes*** ➔ **4.b.** When was the date of the child's most recent **negative** test? __ __/__ __ __ __ *mm yyyy*
- ☐05 Yes, and the test showed that the child does have it ("**positive**" test)
  - ***If yes*** ➔ **4.c.** When was the date of the child's most recent **positive** test? __ __/__ __ __ __ *mm yyyy*

5. Has the child had a blood test to see whether he/she already had the COVID-19 virus ("serology")? (***Mark all that apply***)

- ☐01 No, I never tried to get the child tested
- ☐02 No, I tried to get the child tested but was not able to
- ☐03 Yes, and the child is waiting for the results
  - ***If yes*** ➔ **5.a.** When was the date of the child's most recent test? __ __/__ __ __ __ *mm yyyy*
- ☐04 Yes, and the test showed that the child did not have it ("**negative**" test)
  - ***If yes*** ➔ **5.b.** When was the date of the child's most recent **negative** test? __ __/__ __ __ __ *mm yyyy*
- ☐05 Yes, and the test showed that the child did have it ("**positive**" test)
  - ***If yes*** ➔ **5.c.** When was the date of the child's **positive** test? __ __/__ __ __ __ *mm yyyy*

First we excluded anyone who answered "No, I never tried to get the child tested" to all three questions (remaining N=4,030). Individuals who answered Yes to any of these questions were defined as individuals who were able to procure a test. The remaining individuals were those that answered "No, I tried to get the child tested but was not able to" to at least one of the testing options AND did not endorse yes for any question. They were defined as individuals who were not able to procure a test. For this item, we treated those that were able to get a test as the reference (=0) to specifically highlight disparities in testing access.

Disruptions to health care

Question: In what ways has the COVID-19 outbreak affected the child's overall healthcare? (***Mark all that apply***)

- 01 The child did not go to healthcare appointments because I was concerned about the child entering the healthcare provider's office
- 02 The child's healthcare provider canceled appointments
- 03 The child's healthcare provider changed to phone or online visits
- 04 The child's healthcare provider told him/her to self-isolate or quarantine

© 2023 LeWinn KZ et al. *JAMA Network Open.*

- 05 None of these apply

CODING: Each response option was treated separately as a Yes (=1) or No (=0) variable. If individuals selected more than one option then that individual contributed to the "Yes" group for each item endorsed. Items included in our analysis were: "The child did not go to healthcare appointments because I was concerned about the child entering the healthcare provider's office;" and "The child's health care provider cancelled appointments."

School closure and remote learning supports

School based outcomes were based on the follow portion of the parent-report on child survey:

1. Did the child's school/preschool/daycare close because of the COVID-19 outbreak?

☐01 Yes
☐02 No ➔ ***Skip to Section B, Question 2***
☐03 The child is not enrolled in any school/preschool/daycare ➔ ***Skip to Section B, Question 2***

1.a. Does the child usually receive free meals at school/preschool/daycare?

☐01 Yes
☐02 No ➔ ***Skip to Section B, Question 1.b***

1.a.1. Has the child's school/preschool/daycare offered meals during the closure from COVID-19?

☐01 Yes
☐02 No ➔ ***Skip to Section B, Question 1.b***

1.a.1.a. Has the child been able to get the school-provided meals during the COVID-19 associated closure?

☐01 Yes
☐02 No

1.b. Has the child's school/preschool/daycare offered online learning while closed?

☐01 Yes
☐02 No ➔ ***Skip to Section B, Question 2***

1.b.1. Has the child's school/preschool/daycare provided either of the following to support online learning?

**a.** Free home internet access	☐01 Yes	☐02 No
**b.** Free computer or tablet	☐01 Yes	☐02 No

School Closure
School closure was determined by answers to question 1 above (Yes=1; No=0; 2,768 children not enrolled in school were excluded from this analysis). Note that any child with 01, 02, or 03 endorsed was included in the final analytic sample as children not enrolled in school provided useful information on other pandemic-related experiences.

Continuation of free or reduced price meal service
This outcome was derived by examining answers to questions 1.a. and 1.a.1. Individuals included in this analysis were those that answered yes to 1.a, and 1.a.1. is the item used to derive information on whether schools continued to offer free meals during the pandemic (Yes=1; No=0). 3,998 children contributed to this analysis.

Access to school-provided meals during the COVID-19 associated closures

© 2023 LeWinn KZ et al. *JAMA Network Open.*

This outcome was derived from responses on item 1.a.1.a., and was answered only by those children that answered yes to question 1.a.1 (schools continued to provide school meals during COVID-19 closures). 3,137 children contributed to this analysis.

<u>Schools offered online learning</u>
This outcome was derived by examining all yes endorsements (=1) versus no endorsements (=0) for question 1.b. 9,525 children contributed to this analysis.

<u>Schools provided free home internet</u>
This outcome was derived by examining responses to 1.b.1.a (Yes=1, No=0), which was completed by 7,727 children contributed to this analysis.

<u>Schools provided a free computer/tablet</u>
This outcome was derived by examining responses to 1.b.1.b (Yes=1, No=0), which was completed by 7,767 children contributed to this analysis.

Caregiver Survey Items

Items were derived from the Adult-Report Primary Version of the ECHO COVID-19 surveys. All items were reported by caregivers.

Caregivers were included in the analytic sample if they provided responses for all items below (impacts on work, impacts on childcare, and COVID-19 sources of stress). Caregiver survey items included did not involve any skip logic. All 13,653 participants in the analytic sample contributed responses to these items.

<u>Remote work</u>

Question: In what ways has the COVID-19 outbreak affected your work? (Mark all that apply)

- 01 I moved to working remotely or from home
- 02 I lost my job permanently
- 03 I lost my job temporarily, or was not told for how long
- 04 I got a new job
- 05 I reduced my work hours
- 06 I increased my work hours
- 07 My job put me at increased risk of getting COVID-19
- 08 I laid off employees
- 09 I did not have a paying job before the COVID-19 outbreak
- 10 None of these apply

CODING: Response options were treated separately as endorsed (Yes=1) or not endorsed (No=0). Only the first item (I moved to working remotely or from home) was analyzed (1=Yes; 0=No).

<u>Disruptions to childcare</u>

Question: How has the COVID-19 outbreak affected your regular childcare? (Mark all that apply)

- 01 I had difficulty arranging for childcare
- 02 I had to pay more for childcare
- 03 My spouse/partner or I had to change our work schedule to care for our children ourselves
- 04 My regular childcare has not been affected by the COVID-19 outbreak
- 05 I do not have a child in childcare.

CODING: Each response option was treated separately as a endorsed (Yes=1) or not endorsed (No=0). If individuals selected more than one option then that individual contributed to the "Yes" group for each item endorsed. Only two items were analyzed: 1) I had difficulty arranging childcare, and 2) my spouse/partner or I had to change our work schedule to care for our children ourselves.

<u>Sources of COVID-19-related stress</u>:

Question: What have been your greatest sources of stress from the COVID-19 outbreak? (Mark all that apply)

01 Health concerns

02 Financial concerns

03 Impact on work

04 Impact on your child

05 Impact on your community

06 Impact on family members

07 Access to food

08 Access to baby supplies (e.g., formula, diapers, wipes)

09 Access to personal care products or household supplies

10 Access to medical care, including mental health care

11 Social distancing or being quarantined

12 I am not stressed about the COVID-19 outbreak

CODING: Each response option was treated separately as endorsed (Yes=1) or not endorsed (No=0). If individuals selected more than one option then that individual contributed to the "Yes" group for each item endorsed. All potential stressors were examined except for "I am not stressed about the COVID-19 outbreak."

© 2023 LeWinn KZ et al. *JAMA Network Open.*

eFigure 2. Distribution of COVID-19 surveys over time (A) Child Sample (B) Caregiver Sample

(A)

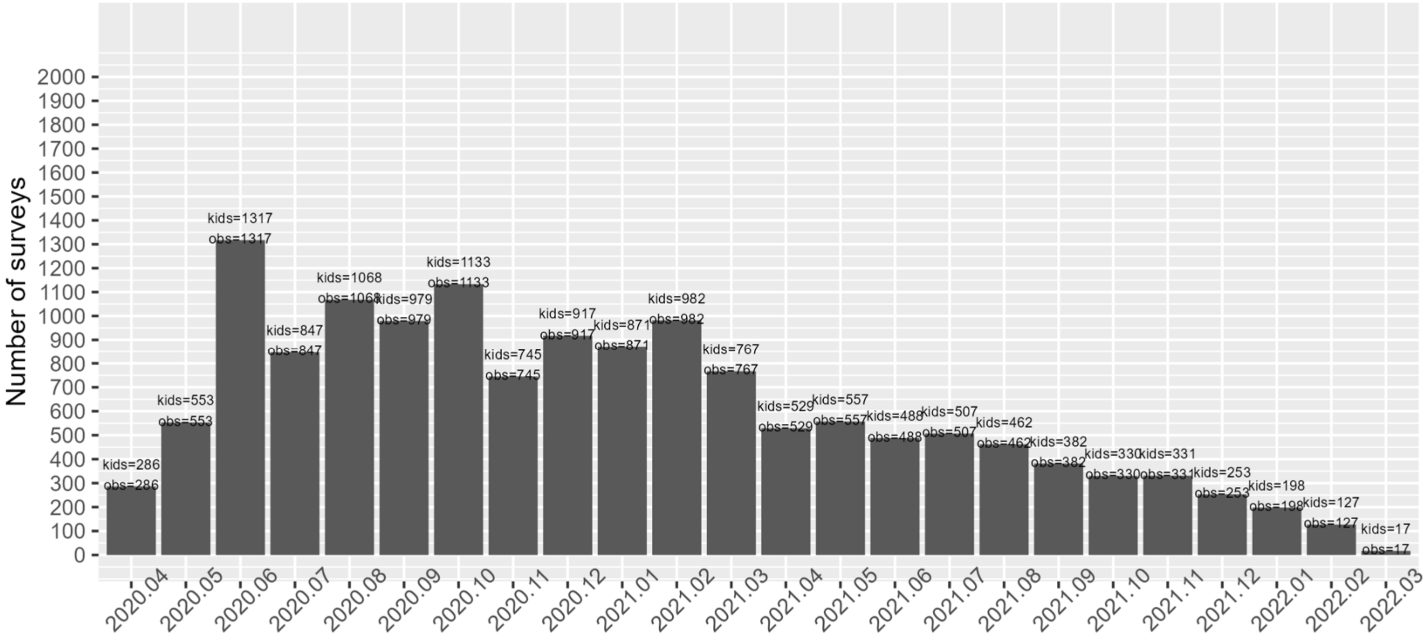

(B)

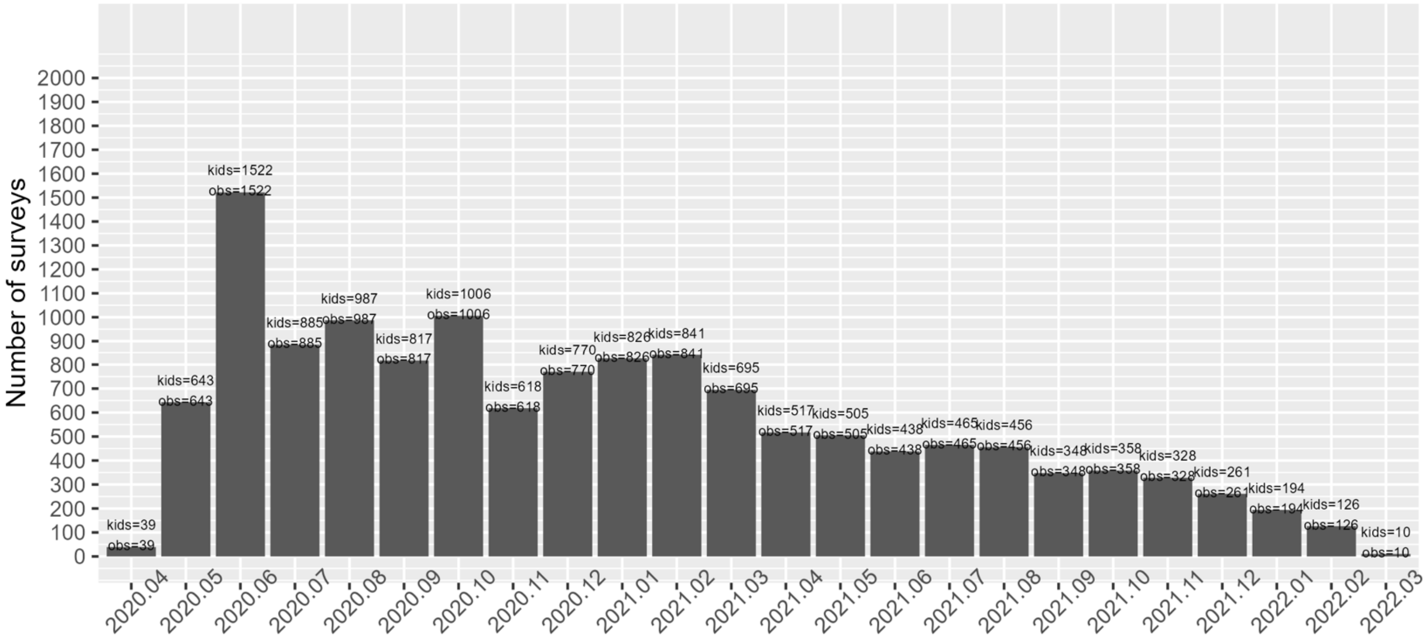

© 2023 LeWinn KZ et al. *JAMA Network Open.*

eTable 1. Differences in Child Health Care, Infection, and Testing by all Exposures of Interest

	Yes, N (%)	No, N (%)	Adjusted OR (95% CI)[a]
**Child didn't get to health care appointment because I was concerned (n=14,646 with data)**			
**Overall**	2,245 (15.3)	12,401 (84.7)	NA
**Maternal education**			
Less than high school	154 (20.0)	615 (80.0)	1.27 (1.02 - 1.58)
High school	326 (17.9)	1,496 (82.1)	1.20 (1.02 - 1.41)
Some college	657 (16.7)	3,267 (83.3)	1.15 (1.01 - 1.32)
Bachelor's degree	541 (12.8)	3,700 (87.2)	0.87 (0.77 - 1.00)
Master's degree and above	567 (14.6)	3,323 (85.4)	reference
**NCHS Urban-Rural Classification**			
Rural	336 (15.6)	1,812 (84.36)	1.12 (0.96 - 1.32)
Urban	1,909 (15.3)	10,589 (84.73)	reference
**Child's life stage at COVID survey completion**			
Middle childhood	867 (13.9)	5,353 (86.06)	0.85 (0.76 - 0.94)
Adolescence	154 (9.2)	1,514 (90.77)	0.50 (0.42 - 0.60)
Early childhood	1,224 (18.1)	5,534 (81.89)	reference
**Provider canceled appointments (n=14,646 with data)**			
**Overall**	2,342 (16.0)	12,304 (84.01)	NA
**Maternal education**			
Less than high school	158 (20.6)	611 (79.45)	1.26 (1.02 - 1.56)
High school	288 (15.8)	1,534 (84.19)	0.99 (0.84 - 1.18)
Some college	624 (15.9)	3,300 (84.1)	1.03 (0.90 - 1.17)
Bachelor's degree	648 (15.3)	3,593 (84.72)	0.96 (0.85 - 1.09)
Master's degree and above	624 (16.0)	3,266 (83.96)	reference
**NCHS Urban-Rural Classification**			
Rural	373 (17.4)	1,775 (82.64)	0.88 (0.75 - 1.02)
Urban	1,969 (15.8)	10,529 (84.25)	reference
**Child's life stage at COVID survey completion**			
Middle childhood	956 (15.4)	5,264 (84.63)	0.99 (0.89 - 1.10)
Adolescence	231 (13.9)	1,437 (86.15)	0.84 (0.71 - 0.98)
Early childhood	1,155 (17.1)	5,603 (82.91)	reference
**Child tested positive for COVID-19 (n=14,646 with data)**			
**Overall**	858 (5.9)	13,788 (94.14)	NA
**Maternal education**			
Less than high school	46 (6.0)	723 (94.02)	0.99 (0.69 - 1.43)
High school	92 (5.1)	1,730 (94.95)	0.97 (0.73 - 1.29)
Some college	263 (6.7)	3,661 (93.3)	1.29 (1.05 - 1.60)
Bachelor's degree	274 (6.5)	3,967 (93.54)	1.42 (1.16 - 1.73)
Master's degree and above	183 (4.7)	3,707 (95.3)	reference
**NCHS Urban-Rural Classification**			
Rural	92 (4.3)	2,056 (95.7)	1.01 (0.78 - 1.29)
Urban	766 (6.1)	11,732 (93.9)	reference
**Child's life stage at COVID survey completion**			
Middle childhood	350 (5.6)	5,870 (94.37)	0.86 (0.73 - 1.01)
Adolescence	87 (5.2)	1,581 (94.78)	0.81 (0.63 - 1.03)
Early childhood	421 (6.2)	6,337 (93.77)	reference
**Unable to get child tested for COVID-19 (n=4,030 with data)**			

© 2023 LeWinn KZ et al. *JAMA Network Open.*

**Overall**	375 (9.3)	3,655 (90.69)	NA
**Maternal education**			
Less than high school	23 (12.5)	161 (87.5)	1.88 (1.06 - 3.33)
High school	68 (15.2)	379 (84.79)	1.97 (1.32 - 2.96)
Some college	104 (9.9)	947 (90.1)	1.33 (0.94 - 1.87)
Bachelor's degree	97 (8.4)	1,065 (91.65)	1.19 (0.86 - 1.65)
Master's degree and above	83 (7.0)	1,103 (93)	reference
	**Yes, N (%)**	**No, N (%)**	**Adjusted OR (95% CI)***
**Unable to get child tested for COVID-19 con't (n=4,030 with data)**			
**NCHS Urban-Rural Classification**			
Urban	45 (11.5)	347 (88.52)	1.13 (0.74 - 1.71)
Rural	330 (9.1)	3,308 (90.9)	reference
**Child's life stage at COVID survey completion**			
Middle childhood	178 (10.0)	1,605 (90.0)	1.11 (0.85 - 1.44)
Adolescence	20 (3.9)	499 (96.2)	0.42 (0.25 - 0.69)
Early childhood	177 (10.2)	1,551 (89.8)	reference

[a]Models are mutually adjusted for maternal education, urban/rural residence, and child life stage. Covariates included are race, and ethnicity; when surveys were completed in relation to different phases of the pandemic (4/2020-7/2020; 8/2020-11/2020; 12/2020-3/2021; 4/2021-7/2021; 8/2021 or later); and U.S. Census division.

© 2023 LeWinn KZ et al. *JAMA Network Open.*

eTable 2. Differences in Pandemic-related School Experiences by All Exposures of Interest

	Yes, N (%)	No, N (%)	Adjusted OR (95% CI)[a]
**School closure because of outbreak (n=11,878 with data)**			
**Overall**	9,788 (82.4)	2,090 (17.6)	NA
**Maternal education**			
Less than high school	443 (83.9)	85 (16.1)	0.95 (0.72 - 1.26)
High school	1,084 (82.6)	228 (17.4)	0.86 (0.71 - 1.04)
Some college	2,544 (82.0)	557 (18.0)	0.87 (0.75 - 1.00)
Bachelor’s degree	2,907 (82.0)	637 (18.0)	0.91 (0.80 - 1.04)
Master’s degree and above	2,810 (82.8)	583 (17.2)	reference
**Child's life stage at COVID survey completion**			
Middle childhood	5,386 (89.2)	654 (10.8)	4.10 (3.64 - 4.62)
Adolescence	1,267 (83.6)	249 (16.4)	2.34 (1.99 - 2.75)
Early childhood	3,135 (72.5)	1,187 (27.5)	reference
**Child received school meals pre-pandemic (n= 9582 with data)**			
**Overall**	4,016 (41.9)	5,566 (58.1)	NA
**Maternal education**			
Less than high school	364 (83.9)	70 (16.1)	12.9 (9.59 - 17.3)
High school	762 (71.9)	298 (28.1)	7.05 (5.86 - 8.49)
Some college	1,491 (59.4)	1,021 (40.6)	4.65 (4.06 - 5.34)
Bachelor’s degree	883 (30.9)	1,977 (69.1)	1.93 (1.68 - 2.21)
Master’s degree and above	516 (19.0)	2,200 (81.0)	reference
**NCHS Urban-Rural Classification**			
Rural	503 (34.8)	943 (65.2)	1.07 (0.90 - 1.27)
Urban	3,513 (43.2)	4,623 (56.8)	reference
**Child's life stage at COVID survey completion**			
Middle childhood	2,183 (40.7)	3,182 (59.3)	0.91 (0.81 - 1.02)
Adolescence	515 (40.8)	748 (59.2)	0.66 (0.56 - 0.77)
Early childhood	1,318 (44.6)	1,636 (55.4)	reference
**School offered online learning during school closure (n=9,525 with data)**			
**Overall**	8,038 (84.4)	1,487 (15.6)	NA
**Maternal education**			
Less than high school	369 (86.2)	59 (13.8)	1.36 (0.94 - 1.95)
High school	909 (86.7)	140 (13.4)	1.37 (1.06 - 1.77)
Some college	2,188 (87.3)	318 (12.7)	1.39 (1.15 - 1.69)
Bachelor’s degree	2,395 (84.3)	447 (15.7)	1.16 (0.97 - 1.37)
Master’s degree and above	2,177 (80.6)	523 (19.4)	reference
**NCHS Urban-Rural Classification**			
Rural	1,181 (82.7)	247 (17.3)	1.04 (0.81 - 1.33)
Urban	6,857 (84.7)	1,240 (15.3)	reference
**Child's life stage at COVID survey completion**			
Middle childhood	5,174 (96.9)	165 (3.1)	35.5 (29.1 - 43.4)
Adolescence	1,214 (96.5)	44 (3.5)	28.1 (20.4 - 38.6)
Early childhood	1,650 (56.4)	1,278 (43.7)	reference
**School offered free meals during school closure (n=3,998 with data)**			
**Overall**	3,162 (79.1)	836 (20.9)	NA
**Maternal education**			
Less than high school	289 (80.3)	71 (19.7)	1.81 (1.24 - 2.63)
High school	620 (82.1)	135 (17.9)	2.12 (1.55 - 2.89)
Some college	1,218 (81.7)	272 (18.3)	2.01 (1.53 - 2.64)
Bachelor’s degree	685 (78.3)	190 (21.7)	1.65 (1.25 - 2.17)

© 2023 LeWinn KZ et al. *JAMA Network Open.*

Master's degree and above	350 (67.6)	168 (32.4)	reference
	**Yes, N (%)**	**No, N (%)**	**Adjusted OR (95% CI)[a]**
**School offered free meals during school closure (n=3,998 with data)**			
**NCHS Urban-Rural Classification**			
Rural	402 (78.5)	110 (21.5)	1.01 (0.74 - 1.37)
Urban	2,760 (79.2)	726 (20.8)	reference
**Child's life stage at COVID survey completion**			
Middle childhood	1,971 (90.6)	205 (9.4)	7.74 (6.34 - 9.44)
Adolescence	454 (88.5)	59 (11.5)	6.83 (5.00 - 9.33)
Early childhood	737 (56.3)	572 (43.7)	reference
**Able to get free meals during school closure (n=3,137 with data)**			
**Overall**	2,279 (72.7)	858 (27.4)	NA
**Maternal education**			
Less than high school	208 (70.5)	87 (29.5)	0.93 (0.64 - 1.37)
High school	441 (71.9)	172 (28.1)	0.88 (0.64 - 1.22)
Some college	884 (72.8)	330 (27.2)	0.90 (0.66 - 1.22)
Bachelor's degree	487 (72.5)	185 (27.5)	0.87 (0.64 - 1.19)
Master's degree and above	259 (75.5)	84 (24.5)	reference
**NCHS Urban-Rural Classification**			
Rural	328 (82.8)	68 (17.2)	1.71 (1.27 - 2.32)
Urban	1,951 (71.2)	790 (28.8)	reference
**Child's life stage at COVID survey completion**	2,279 (72.7)	858 (27.4)	
Middle childhood	1,425 (73.0)	528 (27.0)	0.83 (0.67 - 1.03)
Adolescence	308 (67.8)	146 (32.2)	0.69 (0.52 - 0.91)
Early childhood	546 (74.8)	184 (25.2)	reference
**School offered free internet during school closure (n=7,727 with data)**			
**Overall**	2,248 (29.1)	5,479 (70.9)	NA
**Maternal education, N (%) with data**			
Less than high school	179 (52.8)	160 (47.2)	3.01 (2.29 - 3.94)
High school	370 (42.4)	502 (57.6)	2.03 (1.68 - 2.44)
Some college	672 (32.2)	1,416 (67.8)	1.40 (1.20 - 1.62)
Bachelor's degree	570 (24.6)	1,744 (75.4)	1.13 (0.98 - 1.31)
Master's degree and above	457 (21.6)	1,657 (78.4)	reference
**NCHS Urban-Rural Classification**			
Rural	302 (25.3)	891 (74.7)	1.16 (0.98 - 1.37)
Urban	1,946 (29.8)	4,588 (70.2)	reference
**Child's life stage at COVID survey completion**	2,248 (29.1)	5,479 (70.9)	
Middle childhood	1,540 (30.9)	3,450 (69.1)	1.97 (1.70 - 2.29)
Adolescence	368 (30.9)	824 (69.1)	1.74 (1.44 - 2.10)
Early childhood	340 (22.0)	1,205 (78.0)	reference
**School offered free tablet or computer during school closure (n=7,767 with data)**			
**Overall**	5,549 (71.4)	2,218 (28.6)	NA
**Maternal education**			
Less than high school	286 (82.4)	61 (17.6)	2.19 (1.57 - 3.05)
High school	702 (80.2)	173 (19.8)	2.17 (1.74 - 2.69)
Some college	1,586 (75.3)	519 (24.7)	1.55 (1.33 - 1.81)
Bachelor's degree	1,596 (68.4)	736 (31.6)	1.16 (1.01 - 1.34)
Master's degree and above	1,379 (65.4)	729 (34.6)	reference
**NCHS Urban-Rural Classification**			
Rural	823 (69.1)	368 (30.9)	1.18 (0.98 - 1.41)

© 2023 LeWinn KZ et al. *JAMA Network Open.*

	Yes, N (%)	No, N (%)	Adjusted OR (95% CI)[a]
Urban	4,726 (71.9)	1,850 (28.1)	reference
	**Yes, N (%)**	**No, N (%)**	**Adjusted OR (95% CI)[a]**
**School offered free tablet or computer during school closure con’t (n=7,767 with data)**			
**Child's life stage at COVID survey completion**			
Middle childhood	3,949 (78.7)	1,071 (21.3)	6.70 (5.81 - 7.72)
Adolescence	942 (78.6)	257 (21.4)	5.01 (4.15 - 6.04)
Early childhood	658 (42.5)	890 (57.5)	reference

[a]Models are mutually adjusted for maternal education, urban/rural residence, and child life stage. Covariates included are race, and ethnicity; when surveys were completed in relation to different phases of the pandemic (4/2020-7/2020; 8/2020-11/2020; 12/2020-3/2021; 4/2021-7/2021; 8/2021 or later); and U.S. Census division.

© 2023 LeWinn KZ et al. *JAMA Network Open.*

eTable 3. Differences in Caregiver Work and Childcare by All Exposures of Interest

	Yes, N (%)	No, N (%)	Adjusted OR (95% CI)[a]
**I moved to working remotely or from home (n=13,655 with data)**			
**Overall**	4,221 (30.9)	9,434 (69.1)	NA
**Caregiver education**			
Less than high school	27 (4.4)	594 (95.7)	0.045 (0.030 - 0.067)
High school	144 (9.2)	1,424 (90.8)	0.098 (0.081 - 0.12)
Some college	595 (17.2)	2,864 (82.8)	0.19 (0.17 - 0.22)
Bachelor's degree	1,370 (34.3)	2,624 (65.7)	0.48 (0.44 - 0.53)
Master's degree and above	2,085 (52.0)	1,928 (48.0)	reference
**NCHS Urban Rural Classification**			
Rural	640 (30.4)	1,465 (69.6)	0.78 (0.69 - 0.89)
Urban	3,581 (31.0)	7,969 (69.0)	reference
**Child's life stage at COVID survey completion**	4,221 (30.9)	9,434 (69.1)	
Infant	310 (24.2)	969 (75.8)	0.76 (0.65 - 0.90)
Middle childhood	1,707 (32.2)	3,589 (67.8)	0.98 (0.89 - 1.09)
Adolescence	718 (30.7)	1,619 (69.3)	0.88 (0.78 - 1.00)
Early childhood	1,486 (31.3)	3,257 (68.7)	reference
**I had difficulty arranging for childcare (n=13,655 with data)**			
**Overall**	2,472 (18.1)	11,183 (81.9)	NA
**Caregiver education**			
Less than high school	88 (14.2)	533 (85.8)	0.44 (0.34 - 0.57)
High school	273 (17.4)	1,296 (82.6)	0.57 (0.48 - 0.69)
Some college	598 (17.3)	2,850 (82.7)	0.67 (0.58 - 0.77)
Bachelor's degree	642 (16.1)	3,359 (84.0)	0.69 (0.61 - 0.78)
Master's degree and above	871 (21.7)	3,145 (78.3)	reference
**NCHS Urban Rural Classification**			
Rural	343 (16.3)	1,763 (83.7)	0.91 (0.78 - 1.07)
Urban	2,129 (18.4)	9,420 (81.6)	reference
**Child's life stage at COVID survey completion**			
Infant	204 (15.9)	1,076 (84.1)	0.62 (0.52 - 0.73)
Middle childhood	949 (17.9)	4,346 (82.1)	0.69 (0.62 - 0.77)
Adolescence	110 (4.7)	2,227 (95.3)	0.14 (0.11 - 0.17)
Early childhood	1,209 (25.5)	3,534 (74.5)	reference
**My spouse/partner or I had to change our work schedule to care for our children ourselves (n=13,655 with data)**			
**Overall**	3,364 (24.6)	10,291 (75.4)	NA
**Caregiver education**			
Less than high school	71 (11.4)	552 (88.6)	0.23 (0.17 - 0.30)
High school	186 (11.9)	1,383 (88.2)	0.23 (0.19 - 0.28)
Some college	547 (15.9)	2,890 (84.1)	0.32 (0.28 - 0.36)
Bachelor's degree	1,013 (25.3)	2,995 (74.7)	0.55 (0.50 - 0.61)
Master's degree and above	1,547 (38.5)	2,471 (61.5)	reference
**NCHS Urban Rural Classification**			
Rural	596 (28.2)	1,515 (71.8)	0.98 (0.85 - 1.12)
Urban	2,768 (24.0)	8,776 (76.0)	reference
**Child's life stage at COVID survey completion**	3,364 (24.6)	10,291 (75.4)	
Infant	243 (19.0)	1,036 (81.0)	0.58 (0.49 - 0.68)
Middle childhood	1,442 (27.2)	3,852 (72.8)	0.80 (0.72 - 0.88)
Adolescence	153 (6.6)	2,184 (93.5)	0.12 (0.10 - 0.14)
Early childhood	1,526 (32.2)	3,219 (67.8)	reference

© 2023 LeWinn KZ et al. *JAMA Network Open.*

[a]Models are mutually adjusted for maternal education, urban/rural residence, and child life stage. Covariates included are race, and ethnicity; when surveys were completed in relation to different phases of the pandemic (4/2020-7/2020; 8/2020-11/2020; 12/2020-3/2021; 4/2021-7/2021; 8/2021 or later); and U.S. Census division.

© 2023 LeWinn KZ et al. *JAMA Network Open.*

eTable 4. Differences in Top Pandemic-related Stressors by All Exposures of Interest (n=13,655 with data)

	Yes, N (%)	No, N (%)	Adjusted OR (95% CI)[a]
**Access to food**			
**Overall**	1,641 (12.0)	12,014 (88.0)	NA
**Caregiver education**			
Less than high school	178 (28.4)	449 (71.6)	4.14 (3.20 - 5.36)
High school	309 (19.8)	1,253 (80.2)	3.05 (2.50 - 3.73)
Some college	577 (16.8)	2,863 (83.2)	2.72 (2.25 - 3.28)
Bachelor’s degree	354 (8.8)	3,656 (91.2)	1.57 (1.31 - 1.88)
Master’s degree and above	223 (5.6)	3,793 (94.5)	reference
**Last known NCHS Urban Rural Classification**			
Rural	247 (11.7)	1,860 (88.3)	1.03 (0.87 - 1.23)
Urban	1,394 (12.1)	10,154 (87.9)	reference
**Child's life stage at COVID survey completion**			
Infant	152 (11.9)	1,127 (88.1)	0.95 (0.78 - 1.17)
Middle childhood	564 (10.7)	4,732 (89.4)	0.94 (0.82 - 1.08)
Adolescence	263 (11.3)	2,074 (88.8)	1.01 (0.85 - 1.19)
Early childhood	662 (14.0)	4,081 (86.0)	reference
**Access to supplies**			
**Overall**	3,577 (26.2)	10,078 (73.8)	NA
**Caregiver education**			
Less than high school	237 (38.1)	385 (61.9)	2.06 (1.69 - 2.51)
High school	544 (34.7)	1,025 (65.3)	1.95 (1.69 - 2.26)
Some college	1,122 (32.6)	2,321 (67.4)	1.85 (1.64 - 2.09)
Bachelor’s degree	908 (22.7)	3,095 (77.3)	1.23 (1.10 - 1.38)
Master’s degree and above	766 (19.1)	3,252 (80.9)	reference
**Last known NCHS Urban Rural Classification**			
Rural	556 (26.4)	1,552 (73.6)	0.98 (0.86 - 1.11)
Urban	3,021 (26.2)	8,526 (73.8)	reference
**Child's life stage at COVID survey completion**			
Infant	397 (31.0)	883 (69.0)	1.25 (1.08 - 1.45)
Middle childhood	1,261 (23.8)	4,035 (76.2)	0.85 (0.77 - 0.94)
Adolescence	527 (22.6)	1,810 (77.5)	0.80 (0.70 - 0.90)
Early childhood	1,392 (29.4)	3,350 (70.7)	reference
**Financial concerns**			
**Overall**	4,697 (34.4)	8,958 (65.6)	NA
**Caregiver education**			
Less than high school	303 (48.6)	321 (51.4)	2.02 (1.67 - 2.44)
High school	739 (47.1)	829 (52.9)	2.20 (1.92 - 2.52)
Some college	1,516 (44.1)	1,924 (55.9)	2.14 (1.92 - 2.38)
Bachelor’s degree	1,190 (29.6)	2,825 (70.4)	1.33 (1.20 - 1.47)
Master’s degree and above	949 (23.7)	3,059 (76.3)	reference
**Last known NCHS Urban Rural Classification**			
Rural	699 (33.1)	1,410 (66.9)	1.04 (0.92 - 1.18)
Urban	3,998 (34.6)	7,548 (65.4)	Reference

© 2023 LeWinn KZ et al. *JAMA Network Open.*

	Yes, N (%)	No, N (%)	Adjusted OR (95% CI)[a]
**Financial concerns (con't)**			
**Child's life stage at COVID survey completion**			
Infant	452 (35.3)	827 (64.7)	0.99 (0.86 - 1.14)
Middle childhood	1,754 (33.1)	3,542 (66.9)	0.98 (0.89 - 1.07)
Adolescence	720 (30.8)	1,617 (69.2)	0.86 (0.76 - 0.96)
Early childhood	1,771 (37.3)	2,972 (62.7)	reference
**Health concerns**			
**Overall**	6,027 (44.1)	7,628 (55.9)	NA
**Caregiver education**			
Less than high school	274 (44.1)	347 (55.9)	0.71 (0.59 - 0.86)
High school	601 (38.3)	968 (61.7)	0.62 (0.54 - 0.71)
Some college	1,494 (43.4)	1,948 (56.6)	0.80 (0.73 - 0.89)
Bachelor's degree	1,762 (44.0)	2,245 (56.0)	0.87 (0.80 - 0.95)
Master's degree and above	1,896 (47.2)	2,120 (52.8)	reference
**Last known NCHS Urban Rural Classification**			
Rural	806 (38.3)	1,300 (61.7)	0.77 (0.69 - 0.86)
Urban	5,221 (45.2)	6,328 (54.8)	reference
**Child's life stage at COVID survey completion**			
Infant	590 (46.1)	689 (53.9)	1.13 (0.99 - 1.29)
Middle childhood	2,201 (41.6)	3,095 (58.4)	0.86 (0.79 - 0.94)
Adolescence	977 (41.8)	1,360 (58.2)	0.89 (0.80 - 0.99)
Early childhood	2,259 (47.6)	2,484 (52.4)	reference
**Social distancing or being quarantined**			
**Overall**	7,082 (51.9)	6,573 (48.1)	NA
**Caregiver education**			
Less than high school	267 (42.7)	358 (57.3)	0.56 (0.46 - 0.67)
High school	622 (39.7)	946 (60.3)	0.50 (0.44 - 0.57)
Some college	1,604 (46.6)	1,836 (53.4)	0.63 (0.57 - 0.70)
Bachelor's degree	2,216 (55.3)	1,795 (44.8)	0.87 (0.79 - 0.95)
Master's degree and above	2,373 (59.2)	1,638 (40.8)	reference
**Last known NCHS Urban Rural Classification**			
Rural	1,067 (50.6)	1,042 (49.4)	0.82 (0.73 - 0.91)
Urban	6,015 (52.1)	5,531 (47.9)	reference
**Child's life stage at COVID survey completion**			
Infant	623 (48.8)	655 (51.3)	0.88 (0.77 - 1.00)
Middle childhood	2,794 (52.8)	2,502 (47.2)	0.91 (0.83 - 0.99)
Adolescence	1,079 (46.2)	1,257 (53.8)	0.72 (0.65 - 0.81)
Early childhood	2,586 (54.5)	2,159 (45.5)	reference
**Impact on child**			
**Overall**	8,467 (62.0)	5,188 (38.0)	NA
**Caregiver education**			
Less than high school	289 (46.5)	332 (53.5)	0.42 (0.34 - 0.50)
High school	753 (47.9)	818 (52.1)	0.44 (0.39 - 0.51)
Some college	2,025 (58.8)	1,418 (41.2)	0.67 (0.61 - 0.75)
Bachelor's degree	2,587 (64.6)	1,416 (35.4)	0.82 (0.74 - 0.90)

© 2023 LeWinn KZ et al. *JAMA Network Open.*

Master's degree and above	2,813 (70.0)	1,204 (30.0)	reference
**Impact on child (con't)**			
	**Yes, N (%)**	**No, N (%)**	**Adjusted OR (95% CI)[a]**
**Last known NCHS Urban Rural Classification**			
Rural	1,303 (61.8)	805 (38.2)	0.88 (0.78 - 0.99)
Urban	7,164 (62.0)	4,383 (38.0)	reference
**Child's life stage at COVID survey completion**			
Infant	652 (51.0)	627 (49.0)	0.74 (0.65 - 0.85)
Middle childhood	3,511 (66.3)	1,784 (33.7)	1.21 (1.10 - 1.32)
Adolescence	1,305 (55.9)	1,031 (44.1)	0.73 (0.66 - 0.82)
Early childhood	2,999 (63.2)	1,746 (36.8)	reference
**Impact on community**			
**Overall**	4,430 (32.4)	9,225 (67.6)	NA
**Caregiver education**			
Less than high school	171 (27.5)	452 (72.6)	0.65 (0.53 - 0.79)
High school	369 (23.5)	1,199 (76.5)	0.54 (0.47 - 0.63)
Some college	980 (28.4)	2,468 (71.6)	0.68 (0.61 - 0.76)
Bachelor's degree	1,395 (34.9)	2,604 (65.1)	0.89 (0.81 - 0.98)
Master's degree and above	1,515 (37.7)	2,502 (62.3)	reference
**Last known NCHS Urban Rural Classification**			
Rural	617 (29.3)	1,491 (70.7)	0.78 (0.69 - 0.89)
Urban	3,813 (33.0)	7,734 (67.0)	reference
**Child's life stage at COVID survey completion**			
Infant	376 (29.4)	903 (70.6)	0.95 (0.83 - 1.10)
Middle childhood	1,762 (33.3)	3,534 (66.7)	0.94 (0.86 - 1.03)
Adolescence	698 (29.9)	1,639 (70.1)	0.83 (0.74 - 0.93)
Early childhood	1,594 (33.6)	3,149 (66.4)	reference
**Impact on work**			
**Overall**	4,821 (35.3)	8,834 (64.7)	NA
**Caregiver education**			
Less than high school	203 (32.6)	420 (67.4)	0.56 (0.46 - 0.67)
High school	481 (30.7)	1,085 (69.3)	0.54 (0.47 - 0.62)
Some college	1,128 (32.8)	2,314 (67.2)	0.62 (0.56 - 0.69)
Bachelor's degree	1,315 (32.8)	2,695 (67.2)	0.65 (0.59 - 0.72)
Master's degree and above	1,694 (42.2)	2,320 (57.8)	reference
**Last known NCHS Urban Rural Classification**			
Rural	740 (35.1)	1,368 (64.9)	0.94 (0.83 - 1.05)
Urban	4,081 (35.3)	7,466 (64.7)	reference
**Child's life stage at COVID survey completion**			
Infant	418 (32.7)	859 (67.3)	0.84 (0.73 - 0.97)
Middle childhood	1,899 (35.9)	3,398 (64.2)	0.93 (0.85 - 1.02)
Adolescence	676 (28.9)	1,660 (71.1)	0.67 (0.60 - 0.75)
Early childhood	1,828 (38.5)	2,917 (61.5)	reference

[a]Models are mutually adjusted for maternal education, urban/rural residence, and child life stage. Covariates included are race, and ethnicity; when surveys were completed in relation to different phases of the pandemic (4/2020-7/2020; 8/2020-11/2020; 12/2020-3/2021; 4/2021-7/2021; 8/2021 or later); and U.S. Census division.

© 2023 LeWinn KZ et al. *JAMA Network Open.*
