# Supplementary material for: Sociodemographic Differences in COVID-19 Pandemic Experiences Among Families in the United States
Source: JAMA Netw Open. 2023 Aug 23;6(8):e2330495. doi: 10.1001/jamanetworkopen.2023.30495 (PMC10448300; doi:10.1001/jamanetworkopen.2023.30495)
Supplement: Supplement 2. — Nonauthor Collaborators [file jamanetwopen-e2330495-s002.pdf]

Supplemental Online Content: Nonauthor Collaborators
*First name, last name, and suffix (if applicable) are required and will appear in PubMed.

| *Group Name(s): Environmental influences on Child Health Outcomes consortium | | | | | | | |
|---|---|---|---|---|---|---|---|
| ***First Name and Middle Initial(s)** | ***Last Name** | ***Suffix (eg, Jr, III)** | Academic Degrees | Institution | Location (city, state/province, country) | Role or Contribution, eg, chair, principal investigator | Group (if more than 1 Group listed in the byline) and/or Subgroup (eg, Steering Committee) |
| P B | Smith | | MD, MHS, MPH | Coordinating Center: Duke Clinical Research Institute | Durham, North Carolina, USA | Principal investigator; Coordinating Center | U2COD023375 (Coordinating Center) |
| K L | Newby | | MD | Coordinating Center: Duke Clinical Research Institute | Durham, North Carolina, USA | Principal investigator; Coordinating Center | U2COD023375 (Coordinating Center) |
| Lisa P | Jacobson | | ScD | Data Analysis Center: Johns Hopkins University | Baltimore, Maryland, USA | Principal investigator; ECHO Data Analysis Center | U24D023382 (Data Analysis Center) |
| C B | Parker | | PhD, MSPH | Data Analysis Center: Research Triangle Park Institute | Durham, North Carolina, USA | Principal investigator; ECHO Data Analysis Center | U24D023382 (Data Analysis Center) |
| Richard C | Gershon | | PhD | Person-Reported Outcomes Core: Northwestern University | Evanston, Illinois, USA | Site PI | U24OD023319 (PRO Core) |
| David | Cella | | PhD | Person-Reported Outcomes Core: Northwestern University | Evanston, Illinois, USA | PI; ECHO Pro-Core | U24OD023319 (PRO Core) |
| Susan L | Teitelbaum | | PhD | Icahn School of Medicine at Mount Sinai | New York, NY, USA | ECHO Cohort Principal Investigator | ECHO Cohort UH3OD023320 |
| Annemarie | Stroustrup | | MD | Icahn School of Medicine at Mount Sinai | New York, NY, USA | ECHO Cohort Principal Investigator | ECHO Cohort UH3OD023320 |
| Andrea L | Lampland | | MD | Children’s Hospital and Clinic | Minneapolis, MN, USA | ECHO Cohort Principal Investigator | ECHO Cohort UH3OD023320 |
| Mark L | Hudak | | MD | University of Florida College of Medicine | Jacksonville, FL, USA | ECHO Cohort Principal Investigator | ECHO Cohort UH3OD023320 |
| Lisa K | Washburn | | MD | Wake Forest University School of Medicine | Winston Salem, NC | ECHO Cohort Principal Investigator | ECHO Cohort UH3OD023320 |
| Glorisa J | Canino | | PhD | University of Puerto Rico | San Jaun, PR, | ECHO Cohort Principal Investigator | ECHO Cohort UH3OD023328 |

Supplemental Online Content: Nonauthor Collaborators
*First name, last name, and suffix (if applicable) are required and will appear in PubMed.

| *First Name and Middle Initial(s) | *Last Name | *Suffix (eg, Jr, III) | Academic Degrees | Institution | Location (city, state/province, country) | Role or Contribution, eg, chair, principal investigator | Group (if more than 1 Group listed in the byline) and/or Subgroup (eg, Steering Committee) |
|---|---|---|---|---|---|---|---|
| Steven L | Pastyrnak | | PhD | Helen DeVos Children's Hospital | Grand Rapids, MI, USA | ECHO Cohort Principal Investigator | ECHO Cohort UH3OD023347 |
| Charles | Neal | | MD | Kapiolani Medical Center for Women and Children | Providence, RI, USA | ECHO Cohort Principal Investigator | ECHO Cohort UH3OD023347 |
| Brian S | Carter | | MD | Children's Mercy | Kansas City, MO, USA | ECHO Cohort Principal Investigator | ECHO Cohort UH3OD023347 |
| Jennifer B | Helderman | | MD | Wake Forest University School of Medicine | Winston Salem, NC | ECHO Cohort Principal Investigator | ECHO Cohort UH3OD023347 |
| Hyagriv | Simhan | | MD | University of Pittsburgh Medical Center, Magee Women's Hospital | Pittsburgh, PA, USA | ECHO Cohort Principal Investigator | ECHO Cohort UH3OD023349 |
| Jean | Kerver | | PhD | Michigan State University | East Lansing, MI, USA | ECHO Cohort Principal Investigator | ECHO Cohort UH3OD023285 |
| Charles | Barone | | MD | Henry Ford Health System | Detroit, MI, USA | ECHO Cohort Principal Investigator | ECHO Cohort UH3OD023285 |
| Nigel | Paneth | | MD | Michigan State University | East Lansing, MI, USA | ECHO Cohort Principal Investigator | ECHO Cohort UH3OD023285 |
| Michael R | Elliott | | PhD | University of Michigan | Ann Arbor, MI, USA | ECHO Cohort Principal Investigator | ECHO Cohort UH3OD023285 |
| Susan L | Schantz | | PhD | University of Illinois, Beckman Institute | Urbana, IL, USA | ECHO Cohort Principal Investigator | ECHO Cohort UH3OD023272 |
| Robert M | Silver | | MD | University of Utah | Salt Lake City, UT, USA | ECHO Cohort Principal Investigator | ECHO Cohort UH3OD023249 |
| Rosalind J | Wright | | MD | Icahn School of Medicine at Mount Sinai | New York, NY, USA | ECHO Cohort Principal Investigator | ECHO Cohort UH3OD023337 |
| Michelle | Bosquet-Enlow | | phD | Boston Children's Hospital | Boston MA, USA | ECHO Cohort Principal Investigator | ECHO Cohort UH3OD023337 |
| Alex | Mason | | PhD | University of Tennessee Health Sciences Center | Memphis, Tennessee, USA | Cohort PI | ECHO Cohort UH3OD023271 |
| Frances | Tylavsky | | PhD | University of Tennessee Health Sciences Center | Memphis, Tennessee, USA | Cohort PI | ECHO Cohort UH3OD023271 |

Supplemental Online Content: Nonauthor Collaborators
*First name, last name, and suffix (if applicable) are required and will appear in PubMed.

| *First Name and Middle Initial(s) | *Last Name | *Suffix (eg, Jr, III) | Academic Degrees | Institution | Location (city, state/province, country) | Role or Contribution, eg, chair, principal investigator | Group (if more than 1 Group listed in the byline) and/or Subgroup (eg, Steering Committee) |
|---|---|---|---|---|---|---|---|
| Qi | Zhao | | MD PhD | University of Tennessee Health Sciences Center | Memphis, Tennessee, USA | Cohort PI | ECHO Cohort UH3OD023271 |
| Sheela | Sathyanarayana | | MD | Seattle Children's Research Institute | Seattle, WA | ECHO Cohort Principal Investigator | ECHO Cohort UH3OD023271 |
| Chris | Fussman | | MS | Michigan Department of Health and Human Services | Lansing, MI, USA | ECHO Cohort Principal Investigator | ECHO Cohort UH3OD023285 |
| Shohreh F | Farzan | | PhD | University of Southern California | Los Angeles, California, USA | Cohort PI | ECHO Cohort UH3OD023287 |
| Rima | Habre | | ScD, MSc | University of Southern California | Los Angeles, California, USA | Cohort PI | ECHO Cohort UH3OD023287 |
| Robert S | Tepper | | MD, PhD | Indiana University, Riley Hospital for Children | Indianapolis, Indiana, USA | Cohort PI | ECHO Cohort UH3OD023288 |
| James | Gern | | MD | University of Wisconsin | Madison, Wisconsin, USA | Cohort PI | ECHO Cohort UH3OD023282 |
| Rachel L | Miller | | MD | Columbia University Medical Center | New York, New York, USA | Cohort PI | ECHO Cohort UH3OD023282 |
| Ruby HN | Nguyen | | PhD | University of Minnesota | Minneapolis, MN, USA | ECHO Cohort Principal Investigator | ECHO Cohort UH3OD023271 and UH3OD023282 |
| Judy L | Aschner | | MD | Albert Einstein College of Medicine | Bronx, New York, USA | ECHO Cohort Principal Investigator | ECHO Cohort UH3OD023320 |
| Stephanie L | Merhar | | MD | Cincinnati Children's Hospital Medical Center | Cincinnati, Ohio, USA | ECHO Cohort Principal Investigator | ECHO Cohort UH3OD023320 |
| Paul E | Moore | | MD | Vanderbilt Children's Hospital | Nashville, TN, USA | ECHO Cohort Principal Investigator | ECHO Cohort UH3OD023320 |
| Gloria S | Pryhuber | | MD | University of Rochester Medical Center | Rochester, NY, USA | ECHO Cohort Principal Investigator | ECHO Cohort UH3OD023320 |
| Lynne M | Smith | | MD | Los Angeles Biomedical Research Institute at Harbour-UCLA Medical Center | Los Angeles CA, USA | ECHO Cohort Principal Investigator | ECHO Cohort UH3OD023347 |

Supplemental Online Content: Nonauthor Collaborators
*First name, last name, and suffix (if applicable) are required and will appear in PubMed.

| ***First Name and Middle Initial(s)** | ***Last Name** | ***Suffix (eg, Jr, III)** | Academic Degrees | Institution | Location (city, state/province, country) | Role or Contribution, eg, chair, principal investigator | Group (if more than 1 Group listed in the byline) and/or Subgroup (eg, Steering Committee) |
|---|---|---|---|---|---|---|---|
| Emily S | Barrett | | PhD | University of Rochester Medical Center | Rochester, NY, USA | ECHO Cohort Principal Investigator | ECHO Cohort UH3OD023271 and UH3OD023282 |
| Anne Marie | Reynolds | | MD | University of Buffalo, Jacobson School of Medicine and Biomedical Sciences | Buffalo, NY, USA | ECHO Cohort Principal Investigator | ECHO Cohort UH3OD023320 |
| Lisa | Gatzke-Kopp | | PhD | Pennsylvania State University | University Park, PA, USA | ECHO Cohort Principal Investigator | ECHO Cohort UH3OD023332 |
| Margaret M | Swingler | | PhD | University of North Carolina | Chapel Hill, NC, USA | ECHO Cohort Principal Investigator | ECHO Cohort UH3OD023332 |
| Jonathan M | Mansbach | | MD | Boston Children's Hospital | Boston, MA, USA | ECHO Cohort Principal Investigator | ECHO Cohort UH3OD023253 |
| Jonathan M | Spergel | | MD | Children's Hospital of Philadelphia | Philadelphia, PA, USA | ECHO Cohort Principal Investigator | ECHO Cohort UH3OD023253 |
| Edward M | Zoratti | | MD | Henry Ford Health System | Detroit, MI | ECHO Cohort Principal Investigator | ECHO Cohort UH3OD023282 |
| Casper G | Bendixsen | | PhD | Marshfield Clinic Research Institute | Marshfield, WI, USA | ECHO Cohort Principal Investigator | ECHO Cohort UH3OD023282 |
| Leonard B | Bacharier | | MD | Boston Medical Center | Boston MA, USA | ECHO Cohort Principal Investigator | ECHO Cohort UH3OD023282 |
| George T | O'Connor | | MD | Boston Medical Center | Boston MA, USA | ECHO Cohort Principal Investigator | ECHO Cohort UH3OD023282 |
| Meyer | Kattan | | MD | Children's Hospital of New York | New York, NY, USA | ECHO Cohort Principal Investigator | ECHO Cohort UH3OD023282 |
| Katherine | Rivera-Spoljaric | | MD | Washington University in St Louis | St Louis, MO, USA | ECHO Cohort Principal Investigator | ECHO Cohort UH3OD023282 |
| Christine C | Johnson | | PhD | Henry Ford Health System | Detroit, MI, USA | ECHO Cohort Principal Investigator | ECHO Cohort UH3OD023282 |
| Irva | Hertz-Picciotto | | MD | University of California Davis Mind Institute | Sacramento, CA, USA | ECHO Cohort Principal Investigator | ECHO Cohort UH3OD023365 |
| | | | | | | | |

Supplemental Online Content: Nonauthor Collaborators
*First name, last name, and suffix (if applicable) are required and will appear in PubMed.

| *First Name and Middle Initial(s) | *Last Name | *Suffix (eg, Jr, III) | Academic Degrees | Institution | Location (city, state/province, country) | Role or Contribution, eg, chair, principal investigator | Group (if more than 1 Group listed in the byline) and/or Subgroup (eg, Steering Committee) |
|---|---|---|---|---|---|---|---|
| Daphne | Koinis Mitchell | | MD | Memorial Hospital of Rhode Island | Providence RI, USA | ECHO Cohort Principal Investigator | ECHO Cohort UH3OD023313 |
| Viren | D'Sa | | MD | Memorial Hospital of Rhode Island | Providence RI, USA | ECHO Cohort Principal Investigator | ECHO Cohort UH3OD023313 |
| Dana | Dabelea | | MD | University of Colorado Denver | Denver, CO, USA | ECHO Cohort Principal Investigator | ECHO Cohort UH3OD023248 |
| Sean CL | Deoni | | PhD | Memorial Hospital of Rhode Island | Providence RI, USA | ECHO Cohort Principal Investigator | ECHO Cohort UH3OD023313 |
| Alison E | Hipwell | | PhD | University of Pittsburgh | Pittsburgh, PA, USA | ECHO Cohort Principal Investigator | ECHO Cohort UH3OD023244 |
| Leslie D | Leve | | PhD | Prevention Science Institute, University of Oregon | Eugene, OR, USA | ECHO Cohort Principal Investigator | ECHO Cohort UH3OD023389 |
| Scott T | Weiss | | MD | Brigham and Women's Hospital | Boston, MA, USA | ECHO Cohort Principal Investigator | ECHO Cohort UH3OD023268 |
| Kristen | Lyall | | ScD | Drexel Autism Institute | Philadelphia, PA, USA | ECHO Cohort Principal Investigator | ECHO Cohort UH3OD023342 |
| Heather | Volk | | PhD | Johns Hopkins Bloomberg School of Public Health | Baltimore, Maryland, USA | ECHO Cohort Principal Investigator | ECHO Cohort UH3OD023342 |
| Stephen R | Dager | | MD | University of Washington | Seattle, WA, USA | ECHO Cohort Principal Investigator | ECHO Cohort UH3OD023342 |
| Robert T | Schultz | | PhD | Children's Hospital of Philadelphia - Center for Autism Research | Philadelphia, PA, USA | ECHO Cohort Principal Investigator | ECHO Cohort UH3OD023342 |
| Hyagriv | Simhan | | MD | University of Pittsburgh Medical Center, Magee Women's Hospital | Pittsburgh, PA, USA | ECHO Cohort Principal Investigator | ECHO Cohort UH3OD023349 |
| Rawad | Obeid | | MD | Beaumont Health Medical Center | Royal Oak, MI, USA | ECHO Cohort Principal Investigator | ECHO Cohort UH3OD023348 |
| Caitlin | Rollins | | MD | Boston Children's Hospital | Boston, MA, USA | ECHO Cohort Principal Investigator | ECHO Cohort UH3OD023348 |
| Michael E | Msall | | MD | University of Chicago | Chicago IL, USA | ECHO Cohort Principal Investigator | ECHO Cohort UH3OD023348 |

Supplemental Online Content: Nonauthor Collaborators
*First name, last name, and suffix (if applicable) are required and will appear in PubMed.

| ***First Name and Middle Initial(s)** | ***Last Name** | ***Suffix (eg, Jr, III)** | Academic Degrees | Institution | Location (city, state/province, country) | Role or Contribution, eg, chair, principal investigator | Group (if more than 1 Group listed in the byline) and/or Subgroup (eg, Steering Committee) |
|---|---|---|---|---|---|---|---|
| Michael | O'Shea | | MD | University of North Carolina | Chapel Hill, NC | ECHO Cohort Principal Investigator | ECHO Cohort UH3OD023348 |
| Ruben | Vaidya | | MD | Baystate Children’s Hospital | Springfield, MA, USA | ECHO Cohort Principal Investigator | ECHO Cohort UH3OD023348 |
| John | Meeker | | ScD | University of Michigan | Ann Arbor, MI, USA | ECHO Cohort Principal Investigator | ECHO Cohort UH3OD023251 |
| Federico | Laham | | MD | Arnold Palmer Hospital for Children | Orlando, FL, USA | ECHO Cohort Principal Investigator | ECHO Cohort UH3OD023253 |
| Susan | Wu | | MD | Children's Hospital of Los Angeles | Los Angeles, CA, USA | ECHO Cohort Principal Investigator | ECHO Cohort UH3OD023253 |
| Juan C | Celedón | | MD | Children's Hospital of Pittsburgh of UPMC | Pittsburgh, PA, USA | ECHO Cohort Principal Investigator | ECHO Cohort UH3OD023253 |
| Henry T | Puls | | MD | Children's Mercy Hospital & Clinics | Kansas City, MO, USA | ECHO Cohort Principal Investigator | ECHO Cohort UH3OD023253 |
| Stephen J | Teach | | MD | Children's National Hospital | Washington, DC, USA | ECHO Cohort Principal Investigator | ECHO Cohort UH3OD023253 |
| Stephen C | Porter | | PhD | Cincinnati Children's Hospital and Medical Center | Cincinnati, OH, USA | ECHO Cohort Principal Investigator | ECHO Cohort UH3OD023253 |
| Ilana Y | Waynik | | MD | Connecticut Children's Medical Center | Hartford, CT, USA | ECHO Cohort Principal Investigator | ECHO Cohort UH3OD023253 |
| Sujit | Iyer | | MD | Dell Children's Medical Center of Central Texas | Austin, TX, USA | ECHO Cohort Principal Investigator | ECHO Cohort UH3OD023253 |
| Margaret E | Samuels-Kalow | | MD | Massachusetts General Hospital | Boston, MA, USA | ECHO Cohort Principal Investigator | ECHO Cohort UH3OD023253 |
| Amy D | Thompson | | MD | Nemours Children's Hospital | Wilmington, DE, USA | ECHO Cohort Principal Investigator | ECHO Cohort UH3OD023253 |
| Michelle D | Stevenson | | MD | Norton Children’s Hospital | Louisville, KY, USA | ECHO Cohort Principal Investigator | ECHO Cohort UH3OD023253 |
| Cindy S | Bauer | | MD | Phoenix Children’s Hospital | Phoenix AZ, USA | ECHO Cohort Principal Investigator | ECHO Cohort UH3OD023253 |

Supplemental Online Content: Nonauthor Collaborators
*First name, last name, and suffix (if applicable) are required and will appear in PubMed.

| ***First Name and Middle Initial(s)** | ***Last Name** | ***Suffix (eg, Jr, III)** | Academic Degrees | Institution | Location (city, state/province, country) | Role or Contribution, eg, chair, principal investigator | Group (if more than 1 Group listed in the byline) and/or Subgroup (eg, Steering Committee) |
|---|---|---|---|---|---|---|---|
| Nancy R | Inhofe | | MD | Oklahoma University – Tulsa | Tulsa, OK, USA | ECHO Cohort Principal Investigator | ECHO Cohort UH3OD023253 |
| Markus | Boos | | MD | Seattle Children's Hospital | Seattle, WA, USA | ECHO Cohort Principal Investigator | ECHO Cohort UH3OD023253 |
| Charles G | Macias | | MD | Texas Children's Hospital | Houston, TX, USA | ECHO Cohort Principal Investigator | ECHO Cohort UH3OD023253 |
| Catherine | Monk | | PhD | New York State Psychiatric Institute | New York, NY, USA | ECHO Cohort Principal Investigator | ECHO Cohort UH3OD023328 |
| Jonathan | Posner | | MD | Duke University Department of Psychiatry and Behavioral Sciences | Durham, NC, USA | ECHO Cohort Principal Investigator | ECHO Cohort UH3OD023328 |
| Gurjit | Hershey | | MD | Cincinnati Children’s Hospital Medical Center | Cincinnati, OH, USA | ECHO Cohort Principal Investigator | ECHO Cohort UH3OD023282 |
| Kathryn | Keenan | | PhD | University of Chicago | Chicago, IL, USA | ECHO Cohort Principal Investigator | ECHO Cohort UH3OD023244 |
| Jenae | Neiderhiser | | PhD | Pennsylvania State University | University Park, PA, USA | ECHO Cohort Principal Investigator | ECHO Cohort UH3OD023389 |
| Augusto | Litonjua | | MD | Golisano Children’s Hospital | Rochester, NY, USA | ECHO Cohort Principal Investigator | ECHO Cohort UH3OD023268 |
| Robert | Zeiger | | MD | Kaiser Permanente, Southern California | San Diego, CA, USA | ECHO Cohort Principal Investigator | ECHO Cohort UH3OD023268 |
| Leonard | Bacharier | | MD | Washington University of St. Louis | St Louis, MO, USA | ECHO Cohort Principal Investigator | ECHO Cohort UH3OD023282 |
| Rebecca | Landa | | PhD | Kennedy Krieger Institute | Baltimore, MD, USA | ECHO Cohort Principal Investigator | ECHO Cohort UH3OD023342 |
| Sally | Ozonoff | | PhD | University of California Davis Health, MIND Institute | Sacramento, CA, USA | ECHO Cohort Principal Investigator | ECHO Cohort UH3OD023342 |
| Rebecca | Schmidt | | PhD | University of California Davis Health, MIND Institute | Sacramento, CA, USA | ECHO Cohort Principal Investigator | ECHO Cohort UH3OD023342 |
| Joseph | Piven | | MD | University of North Carolina at Chapel Hill | Chapel Hill, NC | ECHO Cohort Principal Investigator | ECHO Cohort UH3OD023342 |

*First name, last name, and suffix (if applicable) are required and will appear in PubMed.

| ***First Name and Middle Initial(s)** | ***Last Name** | ***Suffix (eg, Jr, III)** | Academic Degrees | Institution | Location (city, state/province, country) | Role or Contribution, eg, chair, principal investigator | Group (if more than 1 Group listed in the byline) and/or Subgroup (eg, Steering Committee) |
|---|---|---|---|---|---|---|---|
| Kelly | Bear | | DO | East Carolina University, Brody School of Medicine | Greenville, NC, USA | ECHO Cohort Principal Investigator | ECHO Cohort UH3OD023348 |
| Madeleine | Lenski | | MS | Michigan State University College of Human Medicine | East Lansing, MI, USA | ECHO Cohort Principal Investigator | ECHO Cohort UH3OD023348 |
| Rachana | Singh | | MD | Tufts University School of Medicine | Boston, MA, USA | ECHO Cohort Principal Investigator | ECHO Cohort UH3OD023348 |
| Jean A | Frazier | | MD | University of Massachusetts Chan Medical School | Worcester, MA, USA | ECHO Cohort Principal Investigator | ECHO Cohort UH3OD023348 |
| Semsa | Gogcu | | MD | Atrium Health Wake Forest Baptist | Winston Salem, NC, USA | ECHO Cohort Principal Investigator | ECHO Cohort UH3OD023348 |
| Angela | Montgomery | | MD | Yale School of Medicine | New Haven, CT, USA | ECHO Cohort Principal Investigator | ECHO Cohort UH3OD023348 |
| Karl C | Kuban | | MD | Boston Medical Center | Boston, MA, USA | ECHO Cohort Principal Investigator | ECHO Cohort UH3OD023348 |
| Laurie M | Douglass | | MD | Boston Medical Center | Boston, MA, USA | ECHO Cohort Principal Investigator | ECHO Cohort UH3OD023348 |
| Hernan | Jara | | PhD | Boston Medical Center | Boston, MA, USA | ECHO Cohort Principal Investigator | ECHO Cohort UH3OD023348 |
| Robert | Joseph | | MD | Boston Medical Center | Boston, MA, USA | ECHO Cohort Principal Investigator | ECHO Cohort UH3OD023348 |
| Douglas | Ruden | | PhD | Wayne State University | Detroit, MI, USA | ECHO Cohort Principal Investigator | ECHO Cohort UH3OD023285 |
| Julie | Herbstman | | PhD | Columbia University Medical Center | New York, NY, USA | ECHO Cohort Principal Investigator | ECHO Cohort UH3OD023290 |
| Tracey | Woodruff | | PhD | University of California, San Francisco | San Francisco, CA | ECHO Cohort Principal Investigator | ECHO Cohort UH3OD023272 |
| Angelo P | Giardino | | MD | University of Utah | Salt Lake City, UT, USA | ECHO Cohort Principal Investigator | ECHO Cohort UH3OD023249 |
| Joseph | Stanford | | MD | University of Utah | Salt Lake City, UT, USA | ECHO Cohort Principal Investigator | ECHO Cohort UH3OD023249 |

Supplemental Online Content: Nonauthor Collaborators
*First name, last name, and suffix (if applicable) are required and will appear in PubMed.

| ***First Name and Middle Initial(s)** | ***Last Name** | ***Suffix (eg, Jr, III)** | Academic Degrees | Institution | Location (city, state/province, country) | Role or Contribution, eg, chair, principal investigator | Group (if more than 1 Group listed in the byline) and/or Subgroup (eg, Steering Committee) |
|---|---|---|---|---|---|---|---|
| Mark | Innocenti | | PhD | Utah State University | Logan, UT, USA | ECHO Cohort Principal Investigator | ECHO Cohort UH3OD023249 |
| Elizabeth | Conradt | | PhD | University of Utah | Salt Lake City, UT, USA | ECHO Cohort Principal Investigator | ECHO Cohort UH3OD023249 |
| Kathi | Huddleston | | PhD | George Mason University | Fairfax, VA, USA | ECHO Cohort Principal Investigator | ECHO Cohort UH3OD023337 |
| Shanna | Swan | | PhD | Icahn School of Medicine at Mount Sinai | New York, NY | ECHO Cohort Principal Investigator | ECHO Cohort UH3OD023305 |
