# Supplementary material for: Sociodemographic Differences in COVID-19 Pandemic Experiences Among Families in the United States
Source: JAMA Netw Open. 2023 Aug 23;6(8):e2330495. doi: 10.1001/jamanetworkopen.2023.30495 (PMC10448300; doi:10.1001/jamanetworkopen.2023.30495)
Supplement: Supplement 3. — Data Sharing Statement [file jamanetwopen-e2330495-s003.pdf]

Data Sharing Statement

LeWinn. Sociodemographic Differences in COVID-19 Pandemic Experiences Among Families in the United States. *JAMA Netw Open*. Published August 23, 2023. doi:10.1001/jamanetworkopen.2023.30495

Data
Data available: Yes
Data types: Deidentified participant data
How to access data: De-identified data from the ECHO Program are available through NICHD's Data and Specimen Hub (DASH) (https://dash.nichd.nih.gov)
When available: With publication

Supporting Documents
Document types: None

Additional Information
Who can access the data: DASH is a centralized resource that allows researchers to access data from various studies via a controlled-access mechanism. Researchers can now request access to these data by creating a DASH account and submitting a Data Request Form. The NICHD DASH Data Access Committee will review the request and provide a response in approximately two to three weeks. Once granted access, researchers will be able to use the data for three years.
Types of analyses: Data will be made available for a specified purpose.
Mechanisms of data availability: Requests are reviewed by the NICHD DASH Data Access Committee.
